# Host specificity of parasitoids (Encyrtidae) toward armored scale insects (Diaspididae): Untangling the effect of cryptic species on quantitative food webs

**DOI:** 10.1002/ece3.4344

**Published:** 2018-07-13

**Authors:** Yao‐Guang Qin, Qing‐Song Zhou, Fang Yu, Xu‐Bo Wang, Jiu‐Feng Wei, Chao‐Dong Zhu, Yan‐Zhou Zhang, Alfried P. Vogler

**Affiliations:** ^1^ Key Laboratory of Zoological Systematics and Evolution Institute of Zoology Chinese Academy of Sciences Beijing China; ^2^ College of Life Sciences University of Chinese Academy of Sciences (UCAS) Beijing China; ^3^ Key Laboratory for Silviculture and Conservation of Ministry of Education Beijing Forestry University Beijing China; ^4^ College of Agriculture Shanxi Agricultural University Shanxi China; ^5^ Department of Life Sciences Natural History Museum London UK; ^6^ Department of Life Sciences Imperial College London Ascot UK

**Keywords:** cryptic species, food web structure, host specificity, molecular species delimitation, specialization index

## Abstract

Host specificity of parasitoids may be measured by various specialization indices to assess the variation of interaction strength among species and the structure of the wider interaction network. However, the conclusions from analyses at the species and network levels may differ, which remains poorly explored. In addition, the recovery of cryptic species of hosts and parasitoids with molecular data may affect the structure of inferred interaction links. We quantified host specificity of hymenopteran parasitoids (family Encyrtidae) on armored scale insects (Hemiptera: Diaspididae) from a wide geographic sampling range across the Chinese Mainland based on both morphological and molecular species delimitation. Mitochondrial COI and nuclear 28S markers detected high cryptic species diversity in the encyrtids and to a lesser degree in the diaspidids, which divided generalist morphospecies into complexes of specialists and generalists. One‐to‐one reciprocal host–parasite links were increased in the molecular data set, but different quantitative species‐level indices produced contrasting estimates of specificity from various one‐to‐multiple and multiple‐to‐multiple host–parasite links. Network indices calculated from DNA‐based species, compared to morphology‐based species definitions, showed lower connectance and generality, but greater specialization and compartmentalization of the interaction network. We conclude that a high degree of cryptic species in host–parasitoid systems refines the true network structure and may cause us overestimating the stability of these interaction webs.

## 1 INTRODUCTION

Host specificity, as one of the most fundamental species traits of parasitoids (Poulin & Keeney, 2008) has been of great interest to ecological and evolutionary biologists (Dyer et al., 2007; Godfray, 1994; Hawkins, 1994; Machado, Robbins, Gilbert, & Herre, 2005; Memmott, Godfray, & Gauld, 1994; Morris, Gripenberg, Lewis, & Roslin, 2014; Novotny & Basset, 2005; Rohde, 1992; Willig, Kaufman, & Stevens, 2013). Insect parasitoids have been commonly included in food web studies (Lafferty, Dobson, & Kuris, 2006), and host specificity has been suggested to have a significant effect on the structure of interaction webs. For instance, specialists decrease the number of interspecific interactions (connectance) and increase network compartmentalization (Van Veen, Müller, Pell, & Godfray, 2008), compared to generalists, which can respond rapidly to changing resource conditions and thus stabilize the food web (Eveleigh et al., 2007). Parasitoid species also have a greater chance to persist after a host loss with increasing connectance (Dunne, Williams, & Martinez, 2002; Estrada, 2007). Thus, network analysis of host–parasitoid relationships is a meaningful tool to understand the role of host specificity in structuring interaction webs.

Analytical approaches of host specificity have advanced from simply counting the number of host and parasitoid species, to characterizing host specificity based on the structure of the links among all participants (Memmott & Godfray, 1994). Quantitative food web analyses describe the overall network structure and the strength of host–parasitoid links at two or more trophic levels (Novotny et al., 2010; Schönrogge & Crawley, 2000; Van Veen et al., 2008). The assessment of host specificity and food web structure relies on the accurate delimitation of species, which may be affected by the existence of unrecognized “cryptic” species within the morphologically distinguishable entities (Wirta et al., 2014). In minute and taxonomically difficult parasitoids, molecular methods for species recognition frequently recover additional subgroups that tend to exhibit narrow specificity of interactions (Burns, Janzen, Hajibabaei, Hallwachs, & Hebert, 2008; Derocles et al., 2016; Li et al., 2010; Smith, Wood, Janzen, Hallwachs, & Hebert, 2007; Smith, Woodley, Janzen, Hallwachs, & Hebert, 2006; Smith et al., 2008), which suggests that host–parasitoid webs are more specialized than previously thought (Hrček & Godfray, 2015). The recognition of cryptic species and their host specificity may also change the general properties of host–parasitoid food webs, for example, reducing connectance (Smith et al., 2011), or increasing compartmentalization due to reduced parasitoid sharing of hosts (Derocles et al., 2014). Kaartinen, Stone, Hearn, Lohse, and Roslin (2010), studying gall‐inducing wasps, were the first to investigate the effects of resolving cryptic species on the structure of a host–parasitoid food web, but despite the greater number of entities and difference in species circumscriptions, they found the overall structure of the interaction web to be largely unchanged. However, the effects of molecular versus morphological species delimitation for the structure of interactions webs remain to be studied in a larger number of host–parasitoid systems sampled from a broader taxonomic and ecological range.

We here report the results of an intensive field‐rearing survey of armored scale insects (Hemiptera: Stenorrhyncha: Diaspididae) and a group of solitary parasitoids (Hymenoptera: Encyrtidae) obtained from these hosts across a wide geographic range in the Chinese Mainland. The Encyrtidae are one of the most speciose groups of parasitoids attacking numerous host insects (Noyes, 2018). Based on counts of the number of hosts, many species of Encyrtidae have wide host ranges, while others exhibit strict host selectivity on obligate hosts (Kapranas & Tena, 2015; Noyes & Hayat, 1994). Recent investigations of endoparasitoids of the genus *Anicetus* revealed high specificity on species of soft‐scale insects (Hemiptera: Coccidae) (Zhang et al., 2011). However, the host specificity on Diaspididae has not been critically tested. Among various other parasitoids reared from the same set of diaspidids, the encyrtid parasitoids obtained in the current study were mostly from the tribe Habrolepidini, which is composed of about 160 known species of about 0.5–2 mm body length (some species in our study are only 0.3 mm). Identification of these species has mainly relied on color patterns on antenna and forewing, or dimensions of antennal segments or setae on the forewing (Noyes & Hayat, 1984; Trjapitzin, 1989). However, their small size and similarity in morphological features generally caused difficulties of distinguishing these species. The hosts in the family Diaspididae are the most species‐rich family of scale insects. They produce a protective sheath under which they feed on the sap of the host plant (Miller & Davidson, 2005). The females are always legless and wingless and exhibit complete fusion of the head, thorax, and abdomen into a flattened saclike body, limiting their external morphological features. Not surprisingly, some species of Diaspididae were found to be complexes of cryptic species (Campbell, Lawrence, Hudspath, & Gruwell, 2014; Gwiazdowski & Normark, 2014; Gwiazdowski, Vea, Andersen, & Normark, 2011; Vea, Gwiazdowski, & Normark, 2013).

The limited resolving power of morphological analyses raises the possibility that the host specificity of parasitoids and diaspidid hosts is greatly underestimated because subdivided groups remain unrecognized. We therefore used DNA sequences of the mitochondrial COI and nuclear 28S rRNA genes, coupled with various algorithmic species delimitation methods, to test for cryptic diversity in hosts and parasitoids and to assess the specificity of host–parasitoid interactions among the morphologically cryptic subgroups. Several metrics have been proposed to describe the degree of host specificity following different principles, which can focus either on the specific links between hosts and parasitoids or on the properties of the overall network structure. A side‐by‐side comparison of networks constructed under morphological and molecular species delimitation can reveal the specific effects of taxonomic resolution on the structure of interaction webs.

## 2 MATERIALS AND METHODS

### 2.1 Specimen sampling

**Figure 1:**
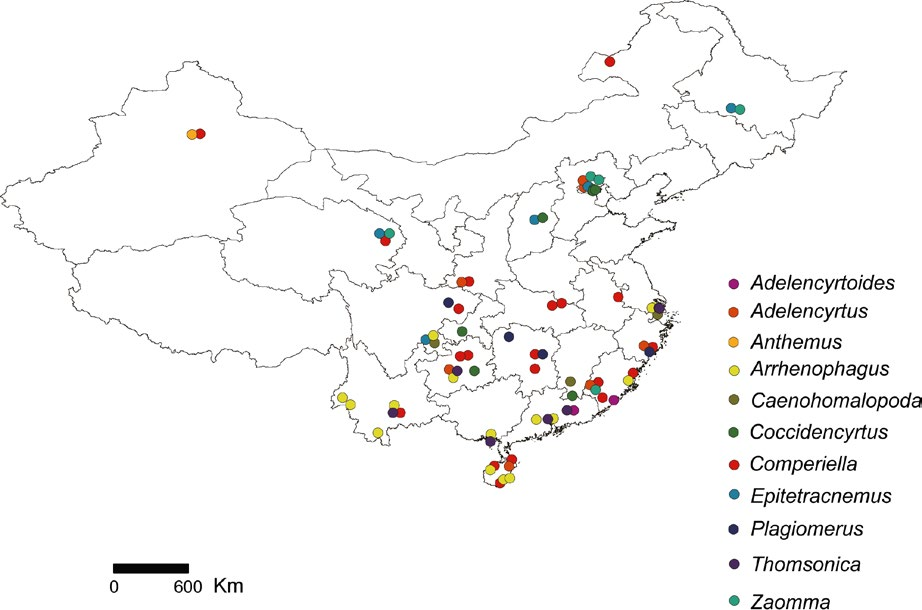
Sampling sites across the Chinese Mainland. Genera of Encyrtidae are shown in different colors

All parasitoids used in this study were reared from adults or late‐stage nymphs of armored scale insects collected in the field across the Chinese Mainland (Figure 1). Most parasitoids were reared from female individuals of the host species except for *Arrhenophagus* spp., which were also reared from males. Host samples (each usually consisting of >500 individuals of a single‐host species) were brought to the laboratory at room temperature. Mixed host species were carefully checked and separated. Each host sample was kept in plastic cups for at least 2 months, and parasitoids were collected immediately when they emerged. The morphological identification of encyrtids and diaspidids was performed by co‐authors YZZ and JFW, respectively, using specialist literature for each group (see Supporting information Data S1). The collected parasitoids and representative host individuals (usually >30) were stored in 95% ethanol for subsequent molecular sequencing. Although many individuals of encyrtid parasitoid were reared from the same unit of host diaspidids, they have a high probability of being siblings and thus only one and five representative individuals from each parasitoid morphospecies and each locality were selected for molecular analysis, as well as the host samples. The sequenced specimens were deposited as vouchers in the Institute of Zoology, Chinese Academy of Sciences, Beijing, China. Detailed information on rearing and origin of the sequenced specimens can be found in Supporting Information Table S1.

### 2.2 DNA extraction, amplification, and sequencing

Genomic DNA extraction was performed using the DNeasy Blood & Tissue Kit (Qiagen GmbH, Hilden, Germany) following the manufacturer's protocols. Amplification of the COI (barcode region) and 28S (D2 region) genes from Encyrtidae used the primer pairs reported in Zhang et al. (2011). In addition, the primer pair FWPTF ‐ LepR1 (Hebert, Penton, Burns, Janzen, & Hallwachs, 2004; Li et al., 2010) was used to generate *a* > 500 bp internal COI sequence for some taxa. The protocols for PCR followed Yu et al. (2014). For Diaspididae, COI sequences were obtained using the primer pairs reported in Morse and Normark (2006) and the 28S sequences (D2 and D3 regions) were obtained using primer 28S‐F3633 and 28S‐b (Rugman‐Jones, Hoddle, & Stouthamer, 2010; Whiting, Carpenter, Wheeler, & Wheeler, 1997). The protocols for PCR followed Wang et al. (2016). PCR products were sequenced using ABI technology. COI sequences were not length‐variable and were aligned using ClustalW, as implemented in BioEdit (Hall, 1999). Translated amino acid sequences were tested for the presence of stop codons using MEGA v 6.0 (Tamura, Stecher, Peterson, Filipski, & Kumar, 2013). The optimal sequence alignment for the length‐variable 28S gene was generated with the iterative algorithm Q‐INS‐i in Mafft v 7.0 (Katoh & Standley, 2013).

### 2.3 Species delimitation

We used three methods of species delimitation: Automatic Barcode Gap Discovery (ABGD); Generalized Mixed Yule‐coalescent (GMYC) (Pons et al., 2006); and Poisson Tree Processes model (PTP). For ABGD, we used the online tool http://wwwabi.snv.jussieu.fr/public/abgd/abgdweb.html, with parameter setting as follows: *P*_min_ = 0.001, *P*_max_ = 0.04, Steps = 50, *X* = 0.75, and Nb bins = 40. ABGD infers species by recursively partitioning the data using a range of prior intraspecific sequence divergences and by calculating a model‐based confidence limit for intraspecific divergence at each iteration (Puillandre, Lambert, Brouillet, & Achaz, 2012; Puillandre, Modica et al., 2012).

The GMYC and PTP analyses were conducted on Bayesian trees obtained with MrBayes v3.1.2 (Ronquist & Huelsenbeck, 2003) for the COI and 28S data separately and the combined dataset. The COI data were partitioned according to the three codon positions. jModelTest selected the GTR+I+G model as the most appropriate model of evolution (Darriba, Taboada, Doallo, & Posada, 2012) for all partitions based on the Akaike information criterion (AIC; Posada & Buckley, 2004). The parameter distributions of Bayesian analyses were checked using Tracer v1.6 (Rambaut, Suchard, Xie, & Drummond, 2013). Searches continued until ESS (effective sample size) for each parameter was larger than 200. The Bayesian phylogenetic trees were made ultrametric using r8s (Sanderson, 2003). The GMYC method infers species boundaries from the transition in branching rate from a Yule (interspecific) to coalescent (intraspecific) model of diversification (Pons et al., 2006). The method was implemented under the single‐threshold setting (Fujisawa & Barraclough, 2013). GMYC analyses were executed in R (R Development Core Team, 2014) using *splits* (from: http://r-forge.r-project.org/projects/splits). PTP analyses equally used the speciation‐to‐coalescent transition of branching rate, but rates were calculated from the number of substitutions between branching events, which avoided the need for ultrametric trees. PTP was performed using the online web server (http://species.h-its.org/ptp/) (Zhang, Kapli, Pavlidis, & Stamatakis, 2013) on the Bayesian tree that was directly supplied as input tree.

### 2.4 Sampling effort assessment

The value of quantitative indices could be strongly dependent on differences in sampling effort (Blüthgen, Menzel, & Blüthgen, 2006; Poisot, Canard, Mouquet, & Hochberg, 2012). We estimated the inventory completeness of the parasitoid community attacking diaspidids using the sample coverage estimator iNEXT online software (Chao, Ma, & Hsieh, 2016), which estimates total species diversity as Simpson diversity from sample size‐based rarefaction and an extrapolation sampling curve.

### 2.5 Network analysis of quantitative food webs

To test how cryptic species of both hosts and parasitoids affect the network analysis of host specificity and food web structure, we combined the results of species delimitation from morphology and molecular analyses to reconstruct four parasitoid–host food webs: (a) diaspidid morphospecies and encyrtid morphospecies (denoted MOR–MOR); (b) diaspidid morphospecies and encyrtid molecular species (MOR–MOL); (c) diaspidid molecular species and encyrtid morphospecies (MOL–MOR); (d) diaspidid molecular species and encyrtid molecular species (MOL–MOL). Networks were constructed on all samples combined obtained from throughout China and separately only the specimens sampled from Yunnan Province at a total of seven sites, as an example of local fauna.

Various species‐level and network‐level indices and network metrics were used to analyze the structure of food webs based on the specific links between hosts and parasitoids, or based on the properties of the overall network structure. Among the species‐focused indices, the Resource Range (RR) is a qualitative index that describes the proportion of host species used by a parasitoid species, as a simple metric for the observed host range (Novotny et al., 2002). Several quantitative indices attempt to capture the strength of interaction links, to account for the difference in host preference of a parasitoid species based on the relative number of individuals with links to particular host species. This can be calculated as the variation of links to the various hosts implemented in the Species Specificity Index (SSI; Julliard, Clavel, Devictor, Jiguet, & Couvet, 2006), or by contrasting the highest link strength to those with all other links, as implemented in the Paired Difference Index (PDI; (Poisot, Lepennetier, Martinez, Ramsayer, & Hochberg, 2010). In addition, the *d*’ (Blüthgen et al., 2006) and Pollination Service Index (PSI; Vázquez, Morris, & Jordano, 2005) take into account the number of links of other species with the host(s) of the focal parasite species, that is, the value is partly dependent on the interactions of the other parasites in the sample. Besides these species‐focused indices, species interactions can be described by their network properties. The most basic level is the number of interspecific interactions (connectance). Nestedness refers to the degree to which the host of ecological specialists overlaps with that of generalists, and modularity is a measure of nonoverlapping subsets of interacting species. Network‐level indices may use binary presence–absence of connections (implemented in Connectance; Blüthgen et al., 2006), in analogy to the qualitative species‐level measures described above. Weighted measures based on the interaction frequency for the calculating the degree of interconnectedness using quantitative indices are implemented in Generality (Dormann, Fründ, Blüthgen, & Gruber, 2009) and *H2*’ (Blüthgen et al., 2006). All analyses were carried out using the bipartite (Dormann et al., 2009) and ESM (Poisot et al., 2012) packages in R (R Development Core Team 2014).

## 3 RESULTS

Standardized sampling of scale insects produced 101 rearing units of >500 individuals of diaspidids parasitized by encyrtids. Morphological examination of 5,775 parasitoid individuals reared from these samples collectively revealed 18 morphospecies belonging to 11 genera of the encyrtid tribe Habrolepidini (Supporting information Table S1). The examination of diaspidid hosts resulted in 28 morphospecies belonging to 13 genera. The estimated rarefaction and extrapolation sampling curve for the Simpson diversity of species and sample coverage indicated that the sample was sufficient to detect most species of parasitoids and diaspidid hosts (Supporting information Figure S1).

**Figure 2:**
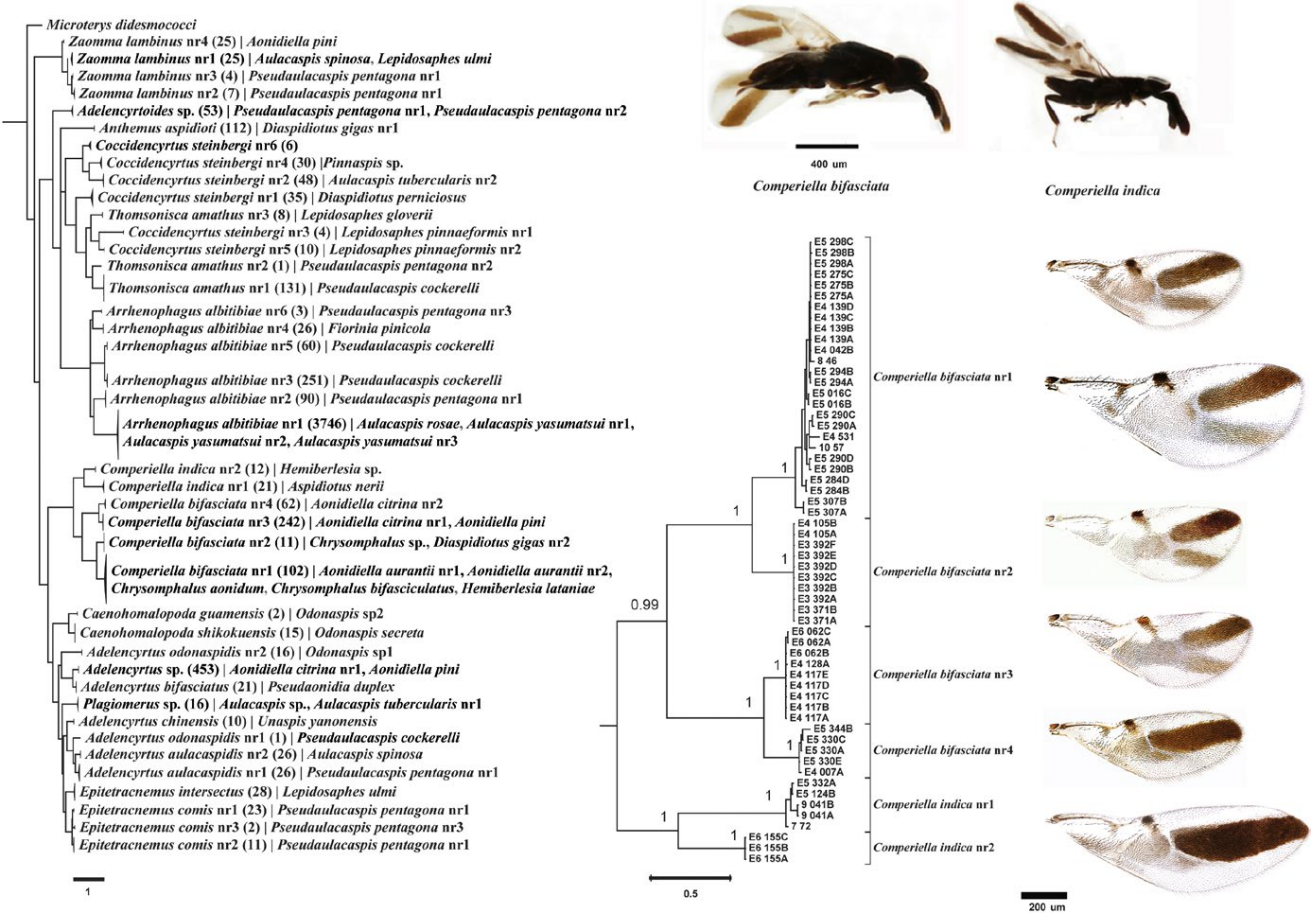
Encyrtidae species delimitation were shown on Bayesian tree inferred using combine dataset of mitochondrial COI and nuclear 28S rRNA, with the outgroup *Microterys didesmococci*. Each one of these 41 twigs represented molecular species encountered among 18 morphospecies. Each encyrtid species name was labeled with the number of encyrtid parasitoid reared from host species in parentheses and host species name. Coccidencyrtus steinbergi nr6 was not obtained confirmative host information. Bayesian tree of representative genus Comperiella was provided, and each of the nodes was supported with high Bayesian posterior probabilities. The following pictures of wings corresponded to each cryptic species of Comperiella from top to bottom

We sequenced 301 representative specimens from all 18 morphologically identified parasitoid species selected to maximize host diversity, which resulted in 253 COI and 291 28S sequences, respectively, including 101 and 56 unique haplotypes. Various species delimitation algorithms applied to the COI, 28S or COI + 28S combined data recovered 41 to 52 entities, of which 6 to 10 were singletons represented by one individual only (Figure 2 and Supporting information Figure S2). Cryptic species were resolved in nine morphospecies: *Adelencyrtus aulacaspidis*,* A*.* odonaspidis*,* Arrhenophagus albitibiae*,* Coccidencyrtus steinbergi*,* Comperiella bifasciata*,* C*. *indica*,* Epitetracnemus comis*,* Thomsonisca amathus*,* Zaomma lambinus*. In virtually all cases, the extent of morphospecies was consistent with the more finely divided COI‐based species, and there were no instances in which different morphospecies were lumped by the COI data. The three algorithms used for species delimitation produced very similar results, and any splitting or lumping did not contradict the extent of entities obtained with the other methods (Table 1). The more conservative 28S marker recovered only about half of these entities, although the conclusions differed between the three algorithmic methods. The 28S‐delimited entities generally matched the morphological delimitation, although in one instance a morphospecies consistent with the COI marker and in another instance closely related morphospecies were lumped into a single entity (Supporting information Figure S2). The newly detected units exhibited COI divergences of at least 3.7% (in *E. comis*) and frequently exceeded 10% (Table 1). In at least one case (*A. odonaspidis*), the two entities were only distantly related. In *C. steinbergi*, the new entities were paraphyletic for *T. amathus* (Figure 2 and Table 1). Intraspecific COI divergences were generally low and showed a maximum of 1.9% in *Z. lambinus* nr1. Molecular species delimitation resulted in a total of 41 species (Table 1 and Supporting information Figure S2).

**Table 1:** Cryptic species revealed by integrating the result of three species delimitation algorithms applied to COI, 28S, and COI+28S combined data

Morphospecies	Entities	K2P divergence	ABGD	GMYC			PTP		
			COI	COI	Combine	28S	COI	Combine	28S
Parasitoid									
*Adelencyrtus aulacaspidis*	2	8.4	y	y + 1	y + 1	n	y	y	n
*Adelencyrtus odonaspidis*	2	n/a	y	y	y	y	y	y	y
*Arrhenophagus albitibiae*	6	6.2–13	y	y + 1	y + 2	y−1	y	y	y−1
*Coccidencyrtus steinbergi*	6	18.7–24.4	y	y + 2	y + 3	y−1	y	y + 1	y−1
*Comperiella bifasciata*	4	5.5–12.4	y	y + 5	y + 2	y + 1	y	y	y
*Comperiella indica*	2	13.9	y	y + 1	y	y	y	y	y
*Epitetracnemus comis*	3	3.7–4.6	y	y	y	y	n	y	n
*Thomsonisca amathus*	3	11.1–12.1	y	y	y	y	y	y	y
*Zaomma lambinus*	4	3.9–10.9	n	y + 2	y + 1	y−1	y	y + 1	n
Host									
*Aonidiella aurantii*	2	6.5	y	y	y	n	y	y + 1	n
*Aonidiella citrina*	2	3.4	y	y	y	n	y	y + 2	n
*Aulacaspis tubercularis*	2	n/a	y	y	y	y	y + 1	y	y
*Aulacaspis yasumatsui*	3	4.0–4.5	y	y	y	y−1	y	y	n
*Diaspidiotus gigas*	2	12.3	y	y	y	y	y	y + 1	n
*Lepidosaphes pinnaeformis*	2	4	y	y	y	n	y	y	n
*Pseudaulacaspis pentagona*	3	5.4–6.3	y	y + 2	y	n	y + 2	y	n

Note

Y: splits are supported by this analysis; n: splits are not supported; n/a: not applicable because the new entities are not sister groups; plus: number of additional entities recovered; minus: number of fewer entities recovered.

Sequences of host diaspidids were obtained from 131 and 138 individuals for COI and 28S, respectively, producing 62 and 48 unique haplotypes. Species delimitation recovered between 25 and 51 entities, of which five to nine were singletons (Table 1 and Supporting information Figure S3). Cryptic species were resolved in seven morphospecies: *Aonidiella aurantii*,* A. citrina*,* Aulacaspis tubercularis*,* A. yasumatsui*,* Diaspidiotus gigas*,* Lepidosaphes pinnaeformis*, and *Pseudaulacaspis pentagona*. Both *A. tubercularis* and *D. gigas* were separated into two cryptic species with interspecies divergence of 11.5% and 12.3%, respectively, and all analysis of ABGD, GMYC, and PTP supported this separation (Table 1 and Supporting information Figure S3). There was no intraspecific variation of 28S in each of five morphospecies. The interspecies divergence of COI revealed apparent separation: each of *A. aurantii*,* A*. *citrina,* and *L. pinnaeformis* consisted of two cryptic species (6.5%, 3.4% and 4.0% respectively); both *A*. *yasumatsui* and *P. pentagona* included three cryptic species (4.0% to 4.5%; 5.4% to 6.3%). All three species delimitation methods generally supported the above result (Supporting information Figure S3). Based on these analyses, molecular species delimitation of diaspidid hosts resulted in 37 species (Supporting information Figure S3).

### 3.1 Impact of cryptic species on specialization and food web structure

**Figure 3:**
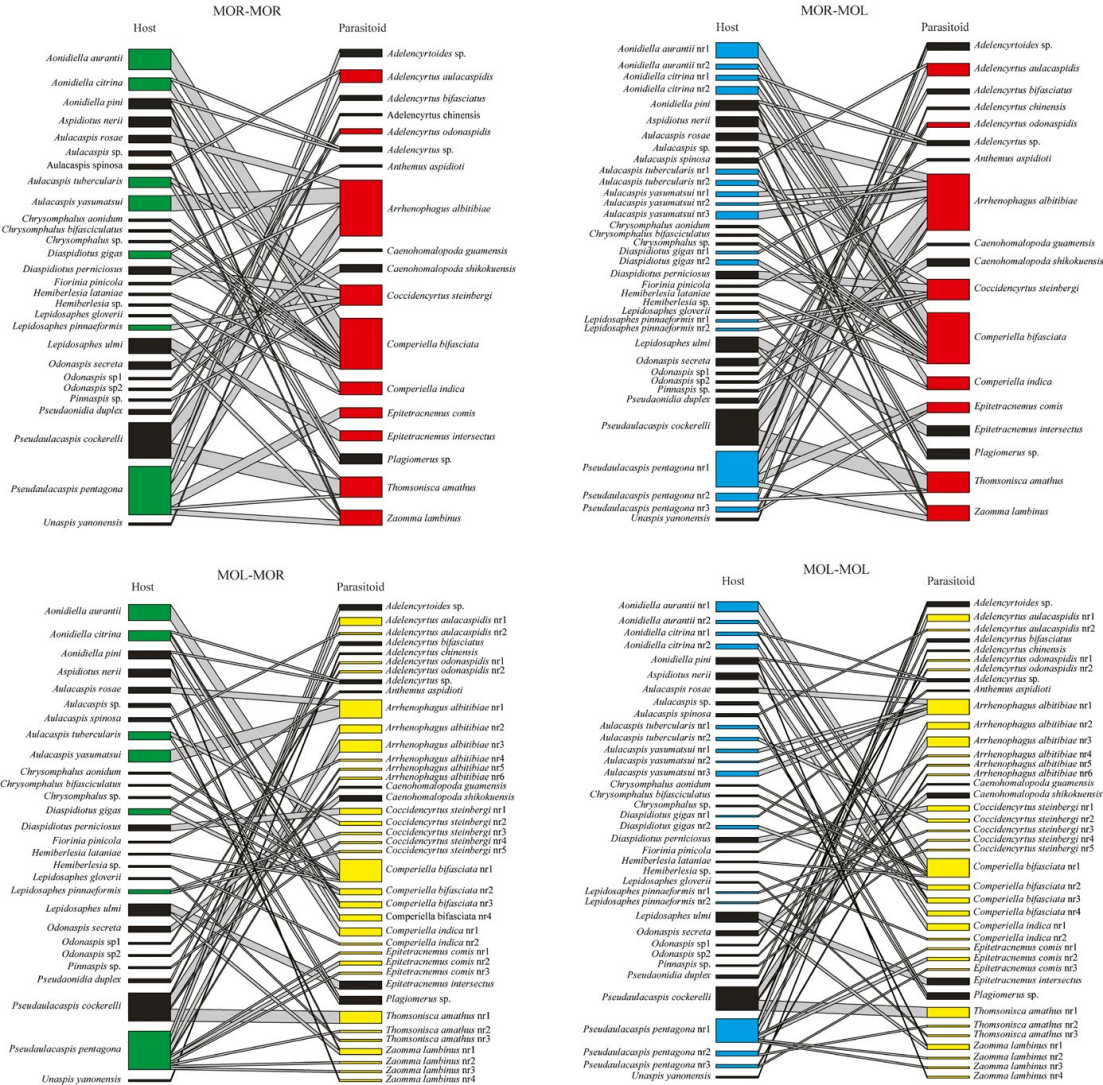
Quantitative food webs reconstructed by crossing the taxonomic results from the morphological and molecular species delimitation. Colored blocks mark the morphospecies of hosts and parasitoids that were split in the molecular analysis: green, Diaspididae morphology; cyan, Diaspididae molecular; red, Encyrtidae morphology; yellow, Encyrtidae molecular

A tanglegram showing host–parasitoid interactions was established for each of the four combinations of morphologically and/or molecularly defined species to assess host use (Figure 3). Overall, there was a limited number of links, with the great majority of parasitoids linked to one or two host species, except for *A. altitibiae* and *C. bifasciata* that used five and eight hosts, respectively. These numbers increased to eight and ten when considering the more finely subdivided molecular host species (MOR–MOL). However, the molecular subdivision of the parasitoid species (MOL–MOR) reduced the number of links to two and four, and to four and five links for the fully molecular species delimitation (MOL–MOL). These two species complexes were the exception to the otherwise high proportion of single‐host specialists, while in all other species the molecular subdivision did not greatly affect the number of hosts.

Applying various metrics for assessing interaction strength, the qualitative index RR reflects the high host specialism (RR = 1.0 or >0.9) for the great majority of species. This proportion increased for the molecularly delimited taxa, which confirmed a high degree of specialism in most cryptic species of parasitoids (Table 2). Compared to RR, quantitative indices of PDI and SSI captured the variation of link strength to detect the differences in degree of specialization among parasitoid utilizing multiple host species. However, the PDI values did not diverge substantially from the qualitative RR, indicating no skew in host use. SSI showed lower values for parasitoids utilizing multiple host species, but these values were similar for parasitoids, showing similar number of host species within and across the four morphological‐molecular datasets and thus indicating rather uniform host use (Table 2).

**Table 2:** Measuring host specificity using various specie‐level indices in four parasitoid–host network

MOR‐MOR	No. of host	RR	PDI	SSI	*d*’	PSI	MOR‐MOL	No. of host	RR	PDI	SSI	*d*’	PSI
*Adelencyrtoides* sp.	1	1.00	1.00	1.00	0.28	0.16	*Epitetracnemus intersectus*	1	1.00	1.00	1.00	0.82	0.67
*Epitetracnemus comis*	1	1.00	1.00	1.00	0.34	0.21	*Adelencyrtus bifasciatus*	1	1.00	1.00	1.00	1.00	1.00
*Anthemus aspidioti*	1	1.00	1.00	1.00	0.63	0.33	*Adelencyrtus chinensis*	1	1.00	1.00	1.00	1.00	1.00
*Epitetracnemus intersectus*	1	1.00	1.00	1.00	0.83	0.67	*Anthemus aspidioti*	1	1.00	1.00	1.00	1.00	1.00
*Adelencyrtus bifasciatus*	1	1.00	1.00	1.00	1.00	1.00	*Caenohomalopoda guamensis*	1	1.00	1.00	1.00	1.00	1.00
*Adelencyrtus chinensis*	1	1.00	1.00	1.00	1.00	1.00	*Caenohomalopoda shikokuensis*	1	1.00	1.00	1.00	1.00	1.00
*Caenohomalopoda guamensis*	1	1.00	1.00	1.00	1.00	1.00	*Adelencyrtus odonaspidis*	2	0.97	0.97	0.70	0.50	0.54
*Caenohomalopoda shikokuensis*	1	1.00	1.00	1.00	1.00	1.00	*Adelencyrtus* sp.	2	0.97	0.97	0.70	0.61	0.38
*Adelencyrtus* sp.	2	0.96	0.96	0.69	0.46	0.23	*Plagiomerus* sp.	2	0.97	0.97	0.70	1.00	1.00
*Adelencyrtus odonaspidis*	2	0.96	0.96	0.69	0.53	0.54	*Adelencyrtoides* sp.	2	0.97	0.99	0.74	0.51	0.47
*Plagiomerus* sp.	2	0.96	0.96	0.69	0.85	0.75	*Epitetracnemus comis*	2	0.97	0.99	0.78	0.40	0.29
*Adelencyrtus aulacaspidis*	2	0.96	0.99	0.82	0.38	0.27	*Adelencyrtus aulacaspidis*	2	0.97	0.99	0.82	0.44	0.33
*Comperiella indica*	2	0.96	0.99	0.82	1.00	1.00	*Comperiella indica*	2	0.97	0.99	0.82	1.00	1.00
*Thomsonisca amathus*	3	0.93	0.99	0.76	0.49	0.45	*Thomsonisca amathus*	3	0.94	0.99	0.76	0.56	0.49
*Zaomma lambinus*	4	0.89	0.93	0.50	0.32	0.27	*Zaomma lambinus*	4	0.92	0.94	0.51	0.30	0.28
*Coccidencyrtus steinbergi*	4	0.89	0.93	0.50	0.91	0.88	*Coccidencyrtus steinbergi*	5	0.89	0.95	0.48	1.00	1.00
*Arrhenophagus albitibiae*	5	0.85	0.92	0.47	0.61	0.67	*Arrhenophagus albitibiae*	8	0.81	0.94	0.40	0.60	0.69
*Comperiella bifasciata*	8	0.74	0.94	0.45	0.89	0.88	*Comperiella bifasciata*	10	0.75	0.94	0.36	0.92	0.93

MOL‐MOR	No. of host	RR	PDI	SSI	*d*’	PSI	MOL‐MOL	No. of host	RR	PDI	SSI	*d*’	PSI
*Arrhenophagus albitibiae* nr6	1	1.00	1.00	1.00	0.00	0.05	*Adelencyrtus odonaspidis* nr1	1	1.00	1.00	1.00	0.00	0.07
*Epitetracnemus comis* nr1	1	1.00	1.00	1.00	0.00	0.05	*Arrhenophagus albitibiae* nr5	1	1.00	1.00	1.00	0.00	0.07
*Epitetracnemus comis* nr3	1	1.00	1.00	1.00	0.00	0.05	*Epitetracnemus comis* nr1	1	1.00	1.00	1.00	0.00	0.07
*Thomsonisca amathus* nr2	1	1.00	1.00	1.00	0.00	0.05	*Zaomma lambinus* nr2	1	1.00	1.00	1.00	0.00	0.07
*Zaomma lambinus* nr2	1	1.00	1.00	1.00	0.00	0.05	*Zaomma lambinus* nr3	1	1.00	1.00	1.00	0.00	0.07
*Zaomma lambinus* nr3	1	1.00	1.00	1.00	0.00	0.05	*Epitetracnemus comis* nr2	1	1.00	1.00	1.00	0.26	0.14
*Adelencyrtus odonaspidis* nr1	1	1.00	1.00	1.00	0.10	0.07	*Adelencyrtus aulacaspidis* nr1	1	1.00	1.00	1.00	0.43	0.29
*Arrhenophagus albitibiae* nr5	1	1.00	1.00	1.00	0.10	0.07	*Arrhenophagus albitibiae* nr2	1	1.00	1.00	1.00	0.43	0.29
*Epitetracnemus comis* nr2	1	1.00	1.00	1.00	0.19	0.11	*Zaomma lambinus* nr4	1	1.00	1.00	1.00	0.47	0.25
*Adelencyrtoides* sp.	1	1.00	1.00	1.00	0.28	0.16	*Arrhenophagus albitibiae* nr3	1	1.00	1.00	1.00	0.56	0.43
*Adelencyrtus aulacaspidis* nr1	1	1.00	1.00	1.00	0.34	0.21	*Thomsonisca amathus* nr1	1	1.00	1.00	1.00	0.56	0.43
*Arrhenophagus albitibiae* nr2	1	1.00	1.00	1.00	0.34	0.21	*Thomsonisca amathus* nr2	1	1.00	1.00	1.00	0.58	0.33
*Zaomma lambinus* nr4	1	1.00	1.00	1.00	0.53	0.25	*Adelencyrtus aulacaspidis* nr2	1	1.00	1.00	1.00	0.74	0.50
*Arrhenophagus albitibiae* nr3	1	1.00	1.00	1.00	0.60	0.43	*Arrhenophagus albitibiae* nr6	1	1.00	1.00	1.00	0.74	0.50
*Thomsonisca amathus* nr1	1	1.00	1.00	1.00	0.60	0.43	*Epitetracnemus comis* nr3	1	1.00	1.00	1.00	0.74	0.50
*Anthemus aspidioti*	1	1.00	1.00	1.00	0.63	0.33	*Epitetracnemus intersectus*	1	1.00	1.00	1.00	0.82	0.67
*Coccidencyrtus steinbergi* nr2	1	1.00	1.00	1.00	0.75	0.50	*Adelencyrtus bifasciatus*	1	1.00	1.00	1.00	1.00	1.00
*Adelencyrtus aulacaspidis* nr2	1	1.00	1.00	1.00	0.76	0.50	*Adelencyrtus chinensis*	1	1.00	1.00	1.00	1.00	1.00
*Coccidencyrtus steinbergi* nr3	1	1.00	1.00	1.00	0.76	0.50	*Adelencyrtus odonaspidis* nr2	1	1.00	1.00	1.00	1.00	1.00
*Coccidencyrtus steinbergi* nr5	1	1.00	1.00	1.00	0.76	0.50	*Anthemus aspidioti*	1	1.00	1.00	1.00	1.00	1.00
*Comperiella bifasciata* nr4	1	1.00	1.00	1.00	0.80	0.60	*Arrhenophagus albitibiae* nr4	1	1.00	1.00	1.00	1.00	1.00
*Epitetracnemus intersectus*	1	1.00	1.00	1.00	0.83	0.67	*Caenohomalopoda guamensis*	1	1.00	1.00	1.00	1.00	1.00
*Adelencyrtus bifasciatus*	1	1.00	1.00	1.00	1.00	1.00	*Caenohomalopoda shikokuensis*	1	1.00	1.00	1.00	1.00	1.00
*Adelencyrtus chinensis*	1	1.00	1.00	1.00	1.00	1.00	*Coccidencyrtus steinbergi* nr1	1	1.00	1.00	1.00	1.00	1.00
*Adelencyrtus odonaspidis* nr2	1	1.00	1.00	1.00	1.00	1.00	*Coccidencyrtus steinbergi* nr2	1	1.00	1.00	1.00	1.00	1.00
*Arrhenophagus albitibiae* nr4	1	1.00	1.00	1.00	1.00	1.00	*Coccidencyrtus steinbergi* nr3	1	1.00	1.00	1.00	1.00	1.00
*Caenohomalopoda guamensis*	1	1.00	1.00	1.00	1.00	1.00	*Coccidencyrtus steinbergi* nr4	1	1.00	1.00	1.00	1.00	1.00
*Caenohomalopoda shikokuensis*	1	1.00	1.00	1.00	1.00	1.00	*Coccidencyrtus steinbergi* nr5	1	1.00	1.00	1.00	1.00	1.00
*Coccidencyrtus steinbergi* nr1	1	1.00	1.00	1.00	1.00	1.00	*Comperiella bifasciata* nr4	1	1.00	1.00	1.00	1.00	1.00
*Coccidencyrtus steinbergi* nr4	1	1.00	1.00	1.00	1.00	1.00	*Comperiella indica* nr1	1	1.00	1.00	1.00	1.00	1.00
*Comperiella indica* nr1	1	1.00	1.00	1.00	1.00	1.00	*Comperiella indica* nr2	1	1.00	1.00	1.00	1.00	1.00
*Comperiella indica* nr2	1	1.00	1.00	1.00	1.00	1.00	*Thomsonisca amathus* nr3	1	1.00	1.00	1.00	1.00	1.00
*Thomsonisca amathus* nr3	1	1.00	1.00	1.00	1.00	1.00	*Adelencyrtus* sp.	2	0.97	0.97	0.70	0.61	0.38
*Adelencyrtus* sp.	2	0.96	0.96	0.69	0.46	0.23	*Plagiomerus* sp.	2	0.97	0.97	0.70	1.00	1.00
*Plagiomerus* sp.	2	0.96	0.96	0.69	0.85	0.75	*Adelencyrtoides* sp.	2	0.97	0.99	0.74	0.51	0.47
*Comperiella bifasciata* nr3	2	0.96	0.98	0.73	0.61	0.40	*Zaomma lambinus* nr1	2	0.97	0.99	0.74	0.59	0.39
*Zaomma lambinus* nr1	2	0.96	0.98	0.73	0.62	0.39	*Comperiella bifasciata* nr3	2	0.97	0.99	0.74	0.71	0.50
*Comperiella bifasciata* nr2	2	0.96	0.98	0.73	0.89	0.78	*Comperiella bifasciata* nr2	2	0.97	0.99	0.74	1.00	1.00
*Arrhenophagus albitibiae* nr1	2	0.96	0.98	0.73	1.00	1.00	*Arrhenophagus albitibiae* nr1	4	0.92	0.94	0.51	1.00	1.00
*Comperiella bifasciata* nr1	4	0.89	0.99	0.73	1.00	1.00	*Comperiella bifasciata* nr1	5	0.89	0.98	0.58	1.00	1.00

Note

MOR: Morphospecies; MOL: Molecular species.

The *d’* and PSI metrics capturing reciprocal specificity with their host species included fewer species with perfect specificity score and showed broader ranges of values overall. This includes several species with *d’ *= 0.0 for the molecularly defined parasitoid species for those with perfect host specificity, that is, RR = 1.0. These are parasitoids of two “superhost” species, *P. cockerelli* and, in particular, *P. pentagona,* which were parasitized by numerous parasitoids, even after being split into multiple units in the molecular analysis. Vice versa, a *d’ *= 1.0 was maintained in some parasitoid species with multiple hosts, that is, RR < 1.0 in the MOL–MOL data, whose multiple hosts in each case have no other parasitoids (Supporting information Table S1). Unraveling cryptic species of parasitoids generally separated one morphospecies into a complex group of parasitoid, each with higher or lower value. Separating cryptic species of hosts increased the value of specialization indices in most parasitoids (Table 2), in particular in the species with the broadest host ranges, *A. albitibiae* and *C. bifasciata*, which were split into six and five species, respectively.

**Figure 4:**
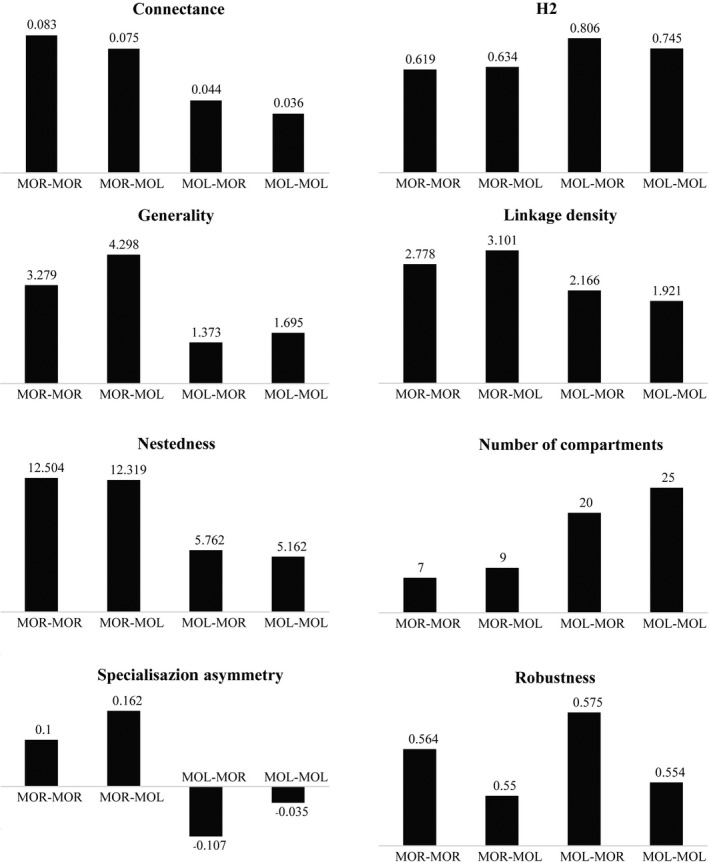
The value of network metrics calculated for these four quantitative food webs in Figure 3

Community‐level parameters were estimated to assess the features of the interaction web. These analyses showed a shift in the web structure when separating the cryptic species of parasitoids, but much less so after separating the cryptic species of hosts (Figures 3 and 4). Connectance, determined as the percentage of the maximum realized links given the total number of host and parasitoid species, was reduced when species were defined by molecular methods, especially for the parasitoids (MOL–MOR and MOL–MOL in Figure 3). The network structure also shifted toward lower value (i.e., more structure) of nestedness (inclusive distribution of specialists among generalists), lower generality (the “effective” mean number of links per parasitoid), lower linkage density (diversity of interactions per species), and lower specialization asymmetry (specialists interacting with generalists). At the same time, compartmentalization (an almost tripling the number of compartments) and specialization within the interaction web, as assessed by H2’ that equals the weighted sum of the specialization of its species described by *d*’ (Blüthgen et al., 2006), were increased. All of these effects were much less evident when separating the cryptic species of the hosts, which only led to a slight decrease in metric values in connectance, nestedness, and number of compartments (Figure 4).

### 3.2 Food web of Yunnan Province

**Figure 5:**
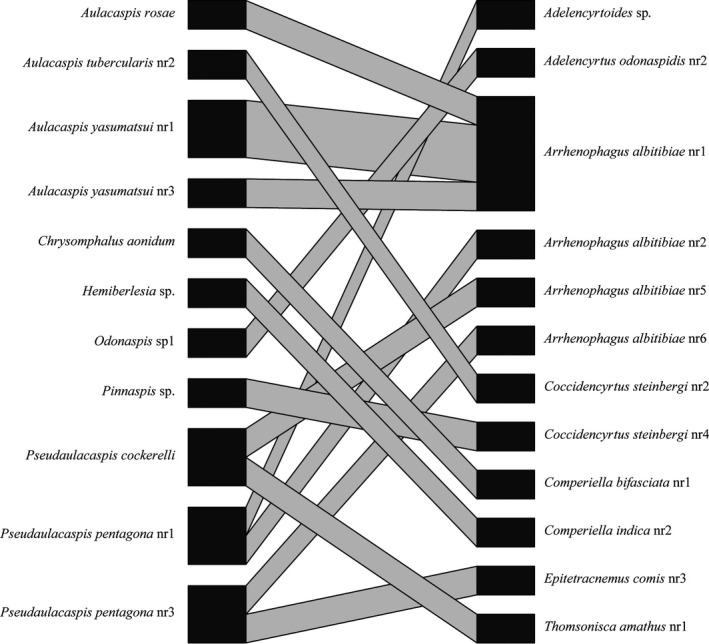
Local food web reconstructed based on all interaction sampled from Yunnan province

The wide geographic scope of the study across the Chinese Mainland did not consider the actual distributional ranges of host and parasitoid species, most of which were limited to some parts of the sampling area. If restricted to a small portion of sites in Yunnan province (Figure 5 and Table 3), compared with the sampling from all of China (MOL–MOL), the network was generally much smaller and showed fewer compartments, composed of 11 host and 12 parasitoid species (compared to 37 and 40 species for the full network). Yet, key features of the network were maintained including the role of species of *Pseudaulacaspis* as the only members hosting multiple parasitoids. In general, the local network showed higher connectance and slightly lower linkage density. The lower H2’ suggested comparatively low specialization, and nestedness was also lower than in the full network, while speciation asymmetry (specialists feeding on generalists) was reduced.

**Table 3:** Values of the network metrics calculated for the comparison of small network (Yunnan) vs. entire network (MOL‐MOL)

Network‐level	Yunnan	MOL‐MOL
Connectance	0.11	0.04
H2	0.5	0.74
Generality	1.49	1.7
Number of compartments	9	25
Nestedness	24.31	5.16
Specialization asymmetry	−0.1	−0.04
Linkage density	1.44	1.92
Robustness	0.55	0.55

## 4 DISCUSSION

We provide an unprecedented empirical data set of host specificity of encyrtid parasitoids reared from scale insects in the family Diaspididae, using both morphological and DNA‐based species delimitation. Despite the involvement of taxonomic specialists providing morphological species circumscriptions of both encyrtid parasitoids and diaspidid hosts, DNA data supported additional subdivision into groupings of high sequence divergence in COI, which were supported by various algorithmic methods for species delineation. The genetically unlinked, conservative 28S marker generally detected the same entities, and where the resolution was not sufficient due to low sequence variation, none of these entities contradicted the morphology‐based or COI‐based species limits. Among the encyrtid parasitoids, nine Linnaean species were split into altogether 32 DNA‐based entities, including one species that was split into six new entities. These findings are in line with other studies, including a threefold increase in the number of genetically delimited *Psyllaephagus* parasitoids over recognized morphospecies (Hall et al., 2017) and the detection of three cryptic species in *Encyrtus sasakii* based on DNA barcoding, geometric morphometrics, and mating tests (Chesters et al., 2012). These cryptic species generally attack a single‐host species, which might suggest that most generalists of encyrtids are complexes of cryptic species with limited host ranges.

This level of partitioning is exacerbated by the detection of cryptic species of the hosts. Cryptic species were discovered in seven of the recognized Linnaean species whose COI sequences split them into altogether 16 species. Diaspididae has been hypothesized to exhibit fine‐scale ecological adaptations, which might result in the separation of multiple cryptic species (Gwiazdowski et al., 2011). This was confirmed, for example, by three cryptic species of *A. nerii* in Australia (Andersen, Gruwell, Morse, & Normark, 2010), while we here detected another two cryptic species in China. Thus, cryptic species diversity of host–parasitoid interactions is much greater than currently recognized.

The greater subdivision of species changed our knowledge of host specificity and network structure. In a specific manner, an increased proportion of species showed extreme specialization and reciprocal interactions with just one partner resulting in values of 1.0 in all species‐level indices. Yet, a number of molecular units retained low *d*’ and PSI indices due to the use of (single) hosts which however acted as host also for other parasitoids. This might be a common feature in host–parasitoid food webs (Cagnolo, Salvo, & Valladares, 2011). When investigating the networks in detail, these low *d’* values largely affect parasitoids using hosts in the genus *Pseudaulacaspis* composed of two Linnaean species, and even after the detection of two cryptic species these newly defined entities each remain the hosts for several parasitoid species. Both *P. cockerelli* (Cockerell scale) and *P. pentagona* (mulberry scale) are widely distributed pests feeding on over 100 different plant species. They are shown here to maintain a disproportional number of parasitoids, which themselves are not supported by any other host (although their cryptic relatives are, in a few cases). Thus, the molecular analysis generally divided the generalist morphospecies into a complex of multiple specialists but also retained a few generalists, as observed in other studies on Aphidiinae (Derocles et al., 2016), Braconidae (Smith et al., 2008) and Tachinidae (Smith et al., 2006, 2007).

The separation of cryptic species had more profound impacts on the network‐level. As suggested by the quantitative network metrics of *H2*’ and Generality, the recognition of the cryptic species tended to increase the degree of specialization of networks, as shown already by the greater proportion of species with high *d*’ and PSI quantitative indices. In addition to this higher level of compartmentalization, the various network parameters agreed that the structure also shifted toward lower number of links (generality) and linkage density (diversity of interactions per species), while the interaction of generalists and specialists was also shifted toward lower value of nestedness and negative value of specialization asymmetry (fewer specialists in parasitoid than host). From an evolutionary perspective, assuming that the Linnaean species generally reflect an earlier node in the phylogeny from which the cryptic species are descended, the more compartmentalized and nested structure in the molecular‐derived network is in agreement with theoretical studies that postulate an evolutionary trend toward greater nestedness in antagonistic interaction webs (Bascompte, Jordano, Melián, & Olesen, 2003). Recent meta‐analyses (Kondoh, Kato, & Sakato, 2010) also supported this hypothesis and suggested nested networks may be a common feature of communities that include resource–consumer interactions. The subdivision of resources among cryptic, specialist species thus supports a mechanism for generating biodiversity based on divergence of host use, consistent with a model of an evolutionary arms race widely assumed in plant–herbivore interactions (Ehrlich & Raven, 1964; Thompson, 2005; Toju et al., 2017).

Yet, one of the striking features of our analysis is that, contrary to the parasitoids, the separation of cryptic species of the hosts has only minor effects on the network structure, as most network parameters in the MOR–MOL networks are little different from MOR‐MOR (Figure 3). This finding in part can be attributed to the lower degree of detected subdivision, which increased from 28 to 37 species in the hosts compared to 18 to 41 in parasitoids, and thus limits how many links are available for altering the network structure. The lack of subdivision might suggest that host specialization is evolutionarily slower and possibly driven by factors other than specialization. It reflects the fact that the hosts also interact with plants and encounter similar issues of antagonistic interactions that may constrain their ability to acquire mechanisms for parasitoid avoidance (that leads to specialization). More detailed analyses of the tritrophic interactions are required to assess the drivers of diversification in diaspidids exerted by the host plant and parasitoids.

At last, the apparent specialization evident from these data has to be seen in the geographic context from where these samples were obtained. Only a small proportion of the investigated species were truly co‐occurring within a single interacting community, unlike in most studies using the specialization indices that are intended to establish the direct interactions among all components of a food web (Kaartinen et al., 2010; Wirta et al., 2014). The network effects of separating cryptic species are therefore overlain by the geographic separation of these entities, perhaps indicating primarily a geographic turnover rather than the evolution of subtly different traits determining host specificity. Our experimental design thus shifts the kinds of questions to be addressed with these network analyses. Each link in the network describes host–parasitoid interactions at some place across a large area (most of mainland China), but as each species of hosts and parasitoids occupies only a certain portion of the study area, these global network interactions appear much more complex than the local networks. This is clearly apparent in the samples reduced to “Yunnan,” which again is not a local community, but the selected records from a subregion within China already provides some insights about the reduced, local networks. The Yunnan network generally detected a higher level of connectivity, indicating that the wider network interactions across China are constrained by the geographic separation, while compartmentalization and nestedness are reduced. Specialization asymmetry (specialists feeding on generalists) also was reduced, supporting the increase in specialism over host generalism at the local level, but it is not clear whether this is due to species‐specific traits or simply the lack of co‐occurrence at a local site. The networks built from the wider sampling regime thus mainly establish the turnover (beta diversity) of host–parasite interactions (in addition to species turnover) across the landscape. For example, the analysis established already that the widespread host species, such as the cosmopolitan *Pseudaulacaspis* species, are attacked by different parasitoids throughout their range. At this stage, the local networks are too poorly sampled to address the interaction beta diversity in detail, but the study sets out such framework for future analyses of more detailed local communities. It also provides information about the kind of parasitoids that attack a widespread species throughout its range, including the analysis of local genetic variants or cryptic species, which may or may not vary in concert in both partners.

## 5 CONCLUSIONS

The use of a China‐wide network, instead of the locally interacting species, provides a framework for addressing the interactions on a biogeographic‐evolutionary level. In addition, as a fundamental trait of parasitoids, the assessment of host specificity mostly depends on our ability to reveal the complexes of cryptic‐species specialist within morphospecies generalists (Derocles et al., 2016; Smith et al., 2008). The impact of interaction strength on assessment of host specificity we observed might therefore revise our conclusions about the relationship between host specificity and food web structure. New molecular methods, including metabarcoding (Yoccoz et al., 2012), could be applied to test much greater numbers of parasitoids, including those of other lineages reared together with the Habrolepidini studied here. Specialization indices can be applied readily to very large datasets for a signature of interaction types, without the detailed inspection of networks conducted here. Thus, the generality of the current findings can now be assessed by studying various group of parasitoids or parasitic organism at various spatial scales.

## CONFLICT OF INTEREST

None declared.

## AUTHOR CONTRIBUTION

YGQ, YZZ, and APV designed the project. YGQ and FY performed laboratory work. YGQ analyzed the data with the help of JFW, FY, QSZ, and XBW. YGQ, YZZ, CDZ, and APV contributed to script development. All authors revised and approved the manuscript.

## DATA ACCESSIBILITY

All new sequences generated in this study have been deposited in GenBank Accession nos.: xx‐yy.
