# Supplementary material for: Host specificity of parasitoids (Encyrtidae) toward armored scale insects (Diaspididae): Untangling the effect of cryptic species on quantitative food webs
Source: Ecol Evol. 2018 Jul 13;8(16):7879–93. doi: 10.1002/ece3.4344 (PMC6144978; doi:10.1002/ece3.4344)
Supplement: Supplementary file 1 [file ECE3-8-7879-s001.pdf]

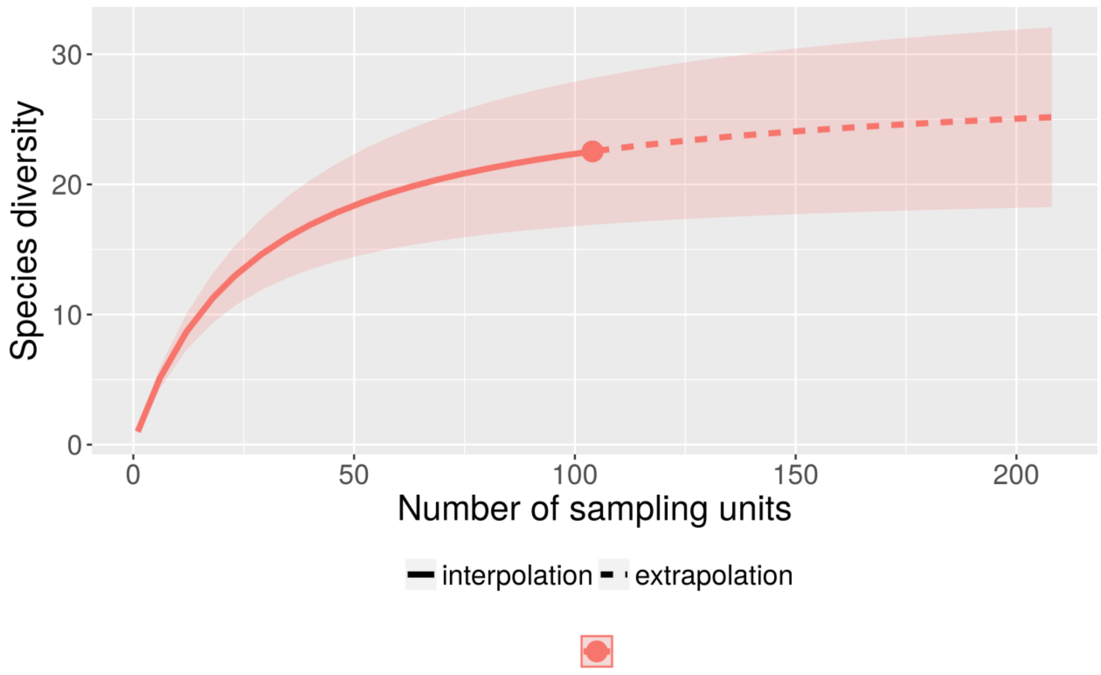

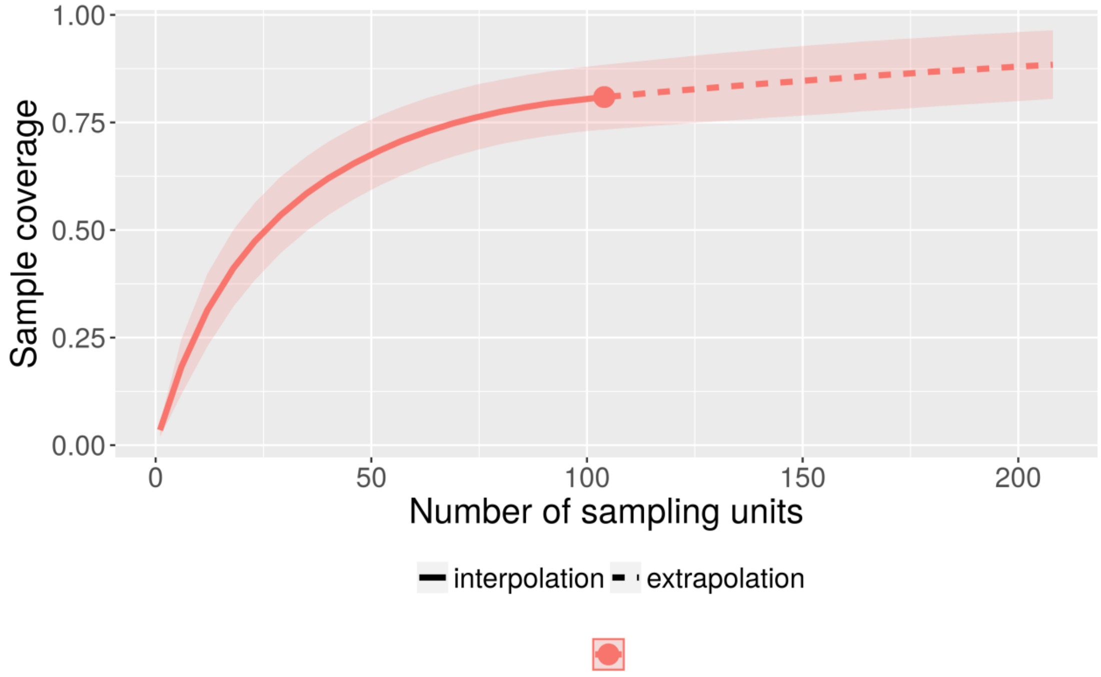

Figure S1 Samplesize-based rarefaction and extrapolation sampling curves for the Simpson diversity of species and samplecoverage throughout the whole collection.
