# Supplementary material for: Host specificity of parasitoids (Encyrtidae) toward armored scale insects (Diaspididae): Untangling the effect of cryptic species on quantitative food webs
Source: Ecol Evol. 2018 Jul 13;8(16):7879–93. doi: 10.1002/ece3.4344 (PMC6144978; doi:10.1002/ece3.4344)
Supplement: Supplementary file 3 [file ECE3-8-7879-s003.pdf]

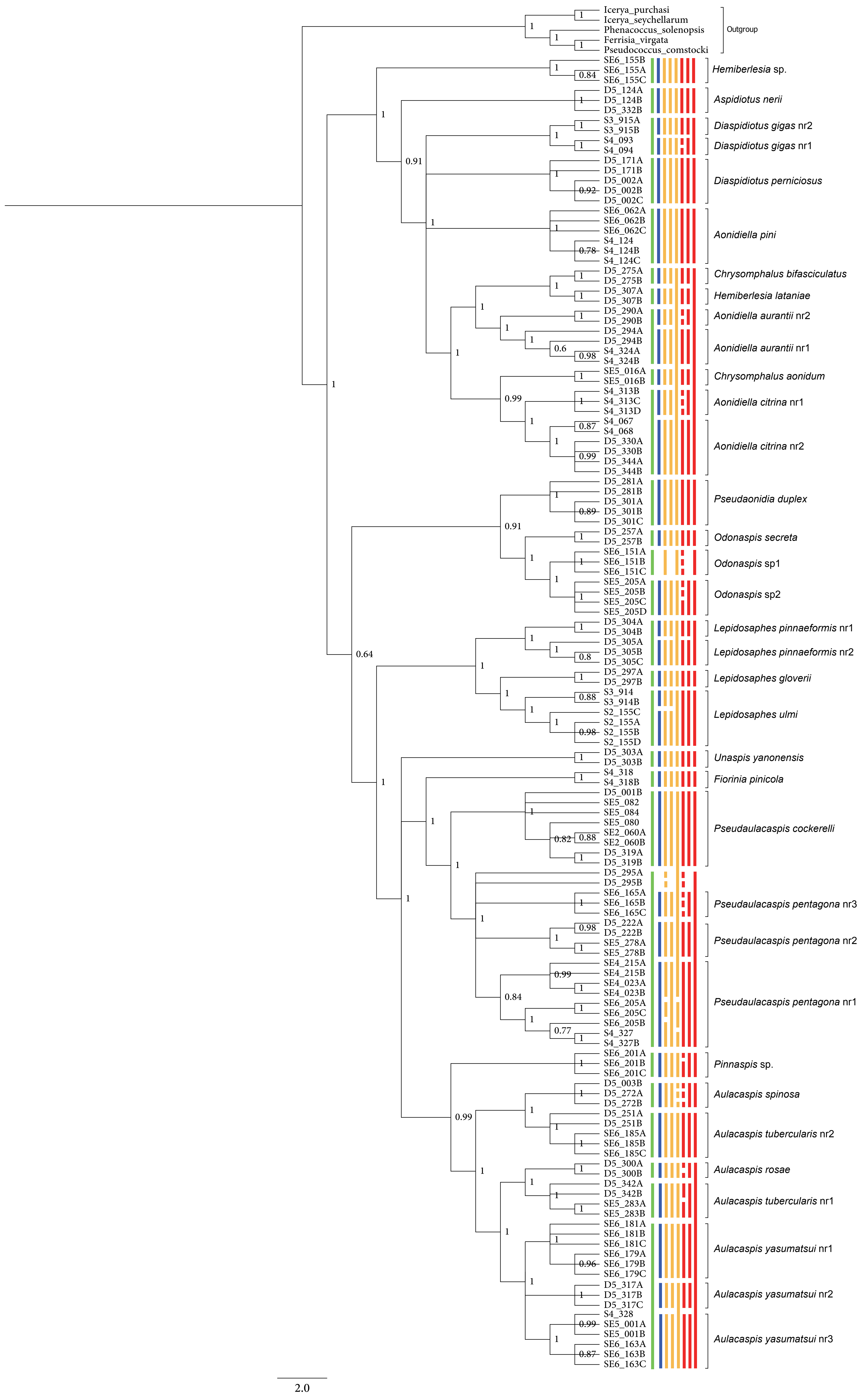

Figure S3 On the left: Bayesian inference phylogeny of Diaspidid host species on the combine dataset. On the right: summary of species delimitation drawn by morphology, ABGD, GMYC (Combine, COI, and 28S), PTP (Combine, COI, and 28S).
