# Supplementary material for: Host specificity of parasitoids (Encyrtidae) toward armored scale insects (Diaspididae): Untangling the effect of cryptic species on quantitative food webs
Source: Ecol Evol. 2018 Jul 13;8(16):7879–93. doi: 10.1002/ece3.4344 (PMC6144978; doi:10.1002/ece3.4344)
Supplement: Supplementary file 4 [file ECE3-8-7879-s004.pdf]

Table S1 Collection Information for all Specimens

Code	Parasitoid	No. reared	Host	Code	Location	Data	Host plant	Collector
10-57	*Comperiella bifasciata* nr1	1	*Aonidiella aurantii* nr1		Hainan, Danzhou	2007.5.11	*Sago cycas*	YZZ
8-46	*Comperiella bifasciata* nr1	1	*Aonidiella aurantii* nr1		Fujian, Nanjing	2008.11.24	*Mangifera indica*	FY
E4-042B	*Comperiella bifasciata* nr1	2	*Aonidiella aurantii* nr1		Fujian, Fuzhou	2014.4.23	*Sago cycas*	JD & YZZ
E4-139A	*Comperiella bifasciata* nr1	6	*Aonidiella aurantii* nr1		Sichuan, Yingshan	2014.8.4	*Citrus maxima*	WXB&JTZ
E4-139B	*Comperiella bifasciata* nr1		*Aonidiella aurantii* nr1	S4-324A	Sichuan, Yingshan	2014.8.4	*Citrus maxima*	WXB&JTZ
E4-139C	*Comperiella bifasciata* nr1		*Aonidiella aurantii* nr1	S4-324B	Sichuan, Yingshan	2014.8.4	*Citrus maxima*	WXB&JTZ
E4-139D	*Comperiella bifasciata* nr1		*Aonidiella aurantii* nr1		Sichuan, Yingshan	2014.8.4	*Citrus maxima*	WXB&JTZ
E5-275A	*Comperiella bifasciata* nr1	19	*Chrysomphalus bifasciculatus*		Fujian, Liancheng	2015.5.15	*Cycas revoluta*	WXB & QSZ
E5-275B	*Comperiella bifasciata* nr1		*Chrysomphalus bifasciculatus*	D5-275A D5-275B	Fujian, Liancheng	2015.5.15	*Cycas revoluta*	WXB & QSZ
E5-275C	*Comperiella bifasciata* nr1		*Chrysomphalus bifasciculatus*		Fujian, Liancheng	2015.5.15	*Cycas revoluta*	WXB & QSZ
E5-294A	*Comperiella bifasciata* nr1	10	*Aonidiella aurantii* nr1		Guizhou, Meitan	2015.7.17	*Photinia serrulata*	JFW
E5-294B	*Comperiella bifasciata* nr1		*Aonidiella aurantii* nr1	D5-294A D5-294B	Guizhou, Meitan	2015.7.17	*Photinia serrulata*	JFW
E5-294C	*Comperiella bifasciata* nr1		*Aonidiella aurantii* nr1		Guizhou, Meitan	2015.7.17	*Photinia serrulata*	JFW
E5-284B	*Comperiella bifasciata* nr1	3	*Aonidiella aurantii* nr1		Zhejiang, Wenzhou	2015.5.20	*Cinnamomum camphora*	WXB & QSZ
E5-284C	*Comperiella bifasciata* nr1		*Aonidiella aurantii* nr1		Zhejiang, Wenzhou	2015.5.20	*Cinnamomum camphora*	WXB & QSZ
E5-284D	*Comperiella bifasciata* nr1		*Aonidiella aurantii* nr1		Zhejiang, Wenzhou	2015.5.20	*Cinnamomum camphora*	WXB & QSZ
E5-290A	*Comperiella bifasciata* nr1	29	*Aonidiella aurantii* nr2		Guizhou, Zunyi	2015.7.17	*Citrus maxima*	JFW
E5-290B	*Comperiella bifasciata* nr1		*Aonidiella aurantii* nr2	D5-290A	Guizhou, Zunyi	2015.7.17	*Citrus maxima*	JFW
E5-290C	*Comperiella bifasciata* nr1		*Aonidiella aurantii* nr2	D5-290B	Guizhou, Zunyi	2015.7.17	*Citrus maxima*	JFW
E5-290D	*Comperiella bifasciata* nr1		*Aonidiella aurantii* nr2		Guizhou, Zunyi	2015.7.17	*Citrus maxima*	JFW
E5-016A	*Comperiella bifasciata* nr1	3	*Chrysomphalus aonidum*		Yunnan, Yuanjiang	2015.2.27	*Ficus microcarpa*	XZ
E5-016B	*Comperiella bifasciata* nr1		*Chrysomphalus aonidum*	SE5-016A SE5-016B	Yunnan, Yuanjiang	2015.2.27	*Ficus microcarpa*	XZ
E5-016C	*Comperiella bifasciata* nr1		*Chrysomphalus aonidum*		Yunnan, Yuanjiang	2015.2.27	*Ficus microcarpa*	XZ
E4-531	*Comperiella bifasciata* nr1	1			Yunnan, XSBN	2014.7.7		
E5-298A	*Comperiella bifasciata* nr1	21	*Aonidiella aurantii* nr2	D5-298A	Guizhou, Guiyang	2015.7.25	*Buxus megistophylla*	WJF
E5-298B	*Comperiella bifasciata* nr1		*Aonidiella aurantii* nr2	D5-298B	Guizhou, Guiyang	2015.7.25	*Buxus megistophylla*	WJF
E5-298C	*Comperiella bifasciata* nr1		*Aonidiella aurantii* nr2		Guizhou, Guiyang	2015.7.25	*Buxus megistophylla*	WJF
E5-307A	*Comperiella bifasciata* nr1	6	*Hemiberlesia lataniae*	D5-307ABCD	Guizhou, Duyun	2015.7.30	*Cycas revoluta*	WJF
E5-307B	*Comperiella bifasciata* nr1		*Hemiberlesia lataniae*		Guizhou, Duyun	2015.7.30	*Cycas revoluta*	WJF
E3-371A	*Comperiella bifasciata* nr2	3	*Diaspidiotus gigas* nr2		Inner Mengolia, Hulun	2013.8.12	*Populus* sp.	
E3-371B	*Comperiella bifasciata* nr2		*Diaspidiotus gigas* nr2		Inner Mengolia, Hulun	2013.8.12	*Populus* sp.	
E3-392A	*Comperiella bifasciata* nr2	6	*Diaspidiotus gigas* nr2		Qinghai, Xining	2013.5.29	*Salix babylonica*	WXB&HBL&XZ
E3-392B	*Comperiella bifasciata* nr2		*Diaspidiotus gigas* nr2		Qinghai, Xining	2013.5.29	*Salix babylonica*	WXB&HBL&XZ
E3-392C	*Comperiella bifasciata* nr2		*Diaspidiotus gigas* nr2	S3-915A	Qinghai, Xining	2013.5.29	*Salix babylonica*	WXB&HBL&XZ
E3-392D	*Comperiella bifasciata* nr2		*Diaspidiotus gigas* nr2	S3-915B	Qinghai, Xining	2013.5.29	*Salix babylonica*	WXB&HBL&XZ
E3-392E	*Comperiella bifasciata* nr2		*Diaspidiotus gigas* nr2		Qinghai, Xining	2013.5.29	*Salix babylonica*	WXB&HBL&XZ
E3-392F	*Comperiella bifasciata* nr2		*Diaspidiotus gigas* nr2		Qinghai, Xining	2013.5.29	*Salix babylonica*	WXB&HBL&XZ
E4-105A	*Comperiella bifasciata* nr2	2	*Chrysomphalus* sp.		Xinjiang, Wulumuqi	2014.5.18	*Populus* sp.	XLT
E4-105B	*Comperiella bifasciata* nr2		*Chrysomphalus* sp.		Xinjiang, Wulumuqi	2014.5.18	*Populus* sp.	XLT
E4-117A	*Comperiella bifasciata* nr3	235	*Aonidiella pini*		Hainan, Haikou	2014.6.7	*Carica papaya*	BC
E4-117B	*Comperiella bifasciata* nr3		*Aonidiella pini*	S4-124	Hainan, Haikou	2014.6.7	*Carica papaya*	BC
E4-117C	*Comperiella bifasciata* nr3		*Aonidiella pini*	S4-124B	Hainan, Haikou	2014.6.7	*Carica papaya*	BC
E4-117D	*Comperiella bifasciata* nr3		*Aonidiella pini*	S4-124C	Hainan, Haikou	2014.6.7	*Carica papaya*	BC
E4-117E	*Comperiella bifasciata* nr3		*Aonidiella pini*		Hainan, Haikou	2014.6.7	*Carica papaya*	BC
E4-128A	*Comperiella bifasciata* nr3	1	*Aonidiella citrina* nr1	S4-313A S4-313B S4-313C S4-313D	Shaanxi, Hanzhong	2014.7.13	*Juncus effusus*	MMN
E6-062A	*Comperiella bifasciata* nr3	6	*Aonidiella pini*	SE6-062A	Hainan, Haikou	2016.8.27	*Plumeria rubra*	YGQ
E6-062B	*Comperiella bifasciata* nr3		*Aonidiella pini*	SE6-062B	Hainan, Haikou	2016.8.27	*Plumeria rubra*	YGQ
E6-062C	*Comperiella bifasciata* nr3		*Aonidiella pini*	SE6-062C	Hainan, Haikou	2016.8.27	*Plumeria rubra*	YGQ
E4-007A	*Comperiella bifasciata* nr4	1	*Aonidiella citrina* nr2	S4-067 S4-068	Fujian, Fuzhou	2014.4.15	*Myrica rubra*	LFP
E5-330A	*Comperiella bifasciata* nr4	50	*Aonidiella citrina* nr2		Hubei, Dawu	2015.10.24	*Podocarpus macrophyllus*	YGQ & JFW
E5-330B	*Comperiella bifasciata* nr4		*Aonidiella citrina* nr2		Hubei, Dawu	2015.10.24	*Podocarpus macrophyllus*	YGQ & JFW
E5-330C	*Comperiella bifasciata* nr4		*Aonidiella citrina* nr2	D5-330A	Hubei, Dawu	2015.10.24	*Podocarpus macrophyllus*	YGQ & JFW
E5-330D	*Comperiella bifasciata* nr4		*Aonidiella citrina* nr2	D5-330B	Hubei, Dawu	2015.10.24	*Podocarpus macrophyllus*	YGQ & JFW
E5-330E	*Comperiella bifasciata* nr4		*Aonidiella citrina* nr2		Hubei, Dawu	2015.10.24	*Podocarpus macrophyllus*	YGQ & JFW
E5-330F	*Comperiella bifasciata* nr4		*Aonidiella citrina* nr2		Hubei, Dawu	2015.10.24	*Podocarpus macrophyllus*	YGQ & JFW
E5-344A	*Comperiella bifasciata* nr4	11	*Aonidiella citrina* nr2		Hunan, Xiangtan	2015.10.26	*Buxus megistophylla*	YGQ & JFW
E5-344B	*Comperiella bifasciata* nr4		*Aonidiella citrina* nr2	D5-344A D5-344B	Hunan, Xiangtan	2015.10.26	*Buxus megistophylla*	YGQ & JFW
E5-344C	*Comperiella bifasciata* nr4		*Aonidiella citrina* nr2		Hunan, Xiangtan	2015.10.26	*Buxus megistophylla*	YGQ & JFW
E5-332A	*Comperiella indica* nr1	5	*Aspidiotus nerii*		Hubei, Dawu	2015.10.30	*Osmanthus fragrans*	YGQ & JFW
E5-332B	*Comperiella indica* nr1		*Aspidiotus nerii*	D5-332A D5-332B	Hubei, Dawu	2015.10.30	*Osmanthus fragrans*	YGQ & JFW
E5-332C	*Comperiella indica* nr1		*Aspidiotus nerii*		Hubei, Dawu	2015.10.30	*Osmanthus fragrans*	YGQ & JFW
E5-124A	*Comperiella indica* nr1	13	*Aspidiotus nerii*		Hunan, Hengyang	2015.4.20	*Osmanthus fragrans*	QSZ & WXB
E5-124B	*Comperiella indica* nr1		*Aspidiotus nerii*	D5-124A D5-124B	Hunan, Hengyang	2015.4.20	*Osmanthus fragrans*	QSZ & WXB
E5-124C	*Comperiella indica* nr1		*Aspidiotus nerii*		Hunan, Hengyang	2015.4.20	*Osmanthus fragrans*	QSZ & WXB
7-72	*Comperiella indica* nr1	1	*Aspidiotus nerii*		Hainan, Diaoluo Mt.	2007.5.6	*Cycas revoluta*	YZZ
9-041A	*Comperiella indica* nr1	2	*Aspidiotus nerii*		Jiangsu, Nanjing	2009.10.9	*Hedera nepalensis*	HLL
9-041B	*Comperiella indica* nr1		*Aspidiotus nerii*		Jiangsu, Nanjing	2009.10.9	*Hedera nepalensis*	HLL
E6-155A	*Comperiella indica* nr2	12	*Hemiberlesia sp.*	SE6-155A	Yunnan, Mengla	2016.10.15	*Ficus microcarpa*	YGQ & XBW
E6-155B	*Comperiella indica* nr2		*Hemiberlesia sp.*	SE6-155B	Yunnan, Mengla	2016.10.15	*Ficus microcarpa*	YGQ & XBW
E6-155C	*Comperiella indica* nr2		*Hemiberlesia sp.*	SE6-155C	Yunnan, Mengla	2016.10.15	*Ficus microcarpa*	YGQ & XBW
E4-116A	*Adelencyrtus* sp.	449	*Aonidiella pini*	S4-124	Hainan, Haikou	2014.6.7	*Carica papaya*	BC
E4-116B	*Adelencyrtus* sp.		*Aonidiella pini*	S4-124B	Hainan, Haikou	2014.6.7	*Carica papaya*	BC
E4-116C	*Adelencyrtus* sp.		*Aonidiella pini*	S4-124C	Hainan, Haikou	2014.6.7	*Carica papaya*	BC
E4-129A	*Adelencyrtus* sp.	4	*Aonidiella citrina* nr1	S4-313A S4-313B	Shaanxi, Hanzhong	2014.7.13	*Juncus effusus*	MMN
E4-129B	*Adelencyrtus* sp.		*Aonidiella citrina* nr1	S4-313C S4-313D	Shaanxi, Hanzhong	2014.7.13	*Juncus effusus*	MMN
E5-281A	*Adelencyrtus bifasciatus*	12	*Pseudaonidia duplex*		Zhejiang, Wenzhou	2015.5.20-6.5	*Camellia japonica*	WXB & QSZ
E5-281B	*Adelencyrtus bifasciatus*		*Pseudaonidia duplex*	D5-281A D5-281B	Zhejiang, Wenzhou	2015.5.20-6.5	*Camellia japonica*	WXB & QSZ
E5-281C	*Adelencyrtus bifasciatus*		*Pseudaonidia duplex*		Zhejiang, Wenzhou	2015.5.20-6.5	*Camellia japonica*	WXB & QSZ
E5-301A	*Adelencyrtus bifasciatus*	9	*Pseudaonidia duplex*	D5-301A	Guizhou, Guiyang	2015.7.27	*Cinnamomum camphora*	JFW&MMN
E5-301B	*Adelencyrtus bifasciatus*		*Pseudaonidia duplex*	D5-301B	Guizhou, Guiyang	2015.7.27	*Cinnamomum camphora*	JFW&MMN
E5-301C	*Adelencyrtus bifasciatus*		*Pseudaonidia duplex*	D5-301C	Guizhou, Guiyang	2015.7.27	*Cinnamomum camphora*	JFW&MMN
E5-319A	*Adelencyrtus odonaspidis* nr1	1	*Pseudaulacaspis cockerelli*	D5-319A	Hainan, Wenchang	2015.10.9	*Trachycarpus fortunei*	CB
E6-151A	*Adelencyrtus odonaspidis* nr2	16	*Odonaspis* sp1	SE6-151A	Yunnan, Jinghong	2016.10.12	*Poaceae*	YGQ & XBW
E6-151B	*Adelencyrtus odonaspidis* nr2		*Odonaspis* sp1	SE6-151B	Yunnan, Jinghong	2016.10.12	*Poaceae*	YGQ & XBW

E6-151C	*Adelencyrtus odonaspidis* nr2		*Odonaspis* sp1	SE6-151C	Yunnan, Jinghong	2016.10.12	*Poaceae*	YGQ & XBW
E6-027A	*Adelencyrtus aulacaspidis* nr1	11	*Pseudaulacaspis pentagona* nr1		Shanxi, Taigu	2016.5.18	*Prunus armeniaca*	YGQ
E6-027B	*Adelencyrtus aulacaspidis* nr1		*Pseudaulacaspis pentagona* nr1		Shanxi, Taigu	2016.5.18	*Prunus armeniaca*	YGQ
E6-027C	*Adelencyrtus aulacaspidis* nr1		*Pseudaulacaspis pentagona* nr1		Shanxi, Taigu	2016.5.18	*Prunus armeniaca*	YGQ
E6-027D	*Adelencyrtus aulacaspidis* nr1		*Pseudaulacaspis pentagona* nr1		Shanxi, Taigu	2016.5.18	*Prunus armeniaca*	YGQ
E4-023C	*Adelencyrtus aulacaspidis* nr1	2	*Pseudaulacaspis pentagona* nr1	SE4-023A	Beijing, Haidian	2014.5.15	*Amygdalus persica*	YW
E4-023D	*Adelencyrtus aulacaspidis* nr1		*Pseudaulacaspis pentagona* nr1	SE4-023B	Beijing, Haidian	2014.5.15	*Amygdalus persica*	YW
E4-025A	*Adelencyrtus aulacaspidis* nr1	5	*Pseudaulacaspis pentagona* nr1		Beijing, Haidian	2014.5.15	*Amygdalus persica*	YW
E4-025B	*Adelencyrtus aulacaspidis* nr1		*Pseudaulacaspis pentagona* nr1		Beijing, Haidian	2014.5.15	*Amygdalus persica*	YW
E5-151A	*Adelencyrtus aulacaspidis* nr1	8	*Pseudaulacaspis pentagona* nr1		Beijing, Yanqing	2015.5.3	*Prunus davidiana*	YW
E5-151B	*Adelencyrtus aulacaspidis* nr1		*Pseudaulacaspis pentagona* nr1		Beijing, Yanqing	2015.5.3	*Prunus davidiana*	YW
E5-151C	*Adelencyrtus aulacaspidis* nr1		*Pseudaulacaspis pentagona* nr1		Beijing, Yanqing	2015.5.3	*Prunus davidiana*	YW
E5-151D	*Adelencyrtus aulacaspidis* nr1		*Pseudaulacaspis pentagona* nr1		Beijing, Yanqing	2015.5.3	*Prunus davidiana*	YW
E5-151E	*Adelencyrtus aulacaspidis* nr1		*Pseudaulacaspis pentagona* nr1		Beijing, Yanqing	2015.5.3	*Prunus davidiana*	YW
E5-272A	*Adelencyrtus aulacaspidis* nr2	26	*Aulacaspis spinosa*	D5-272A	Fujian, Liancheng	2015.5.3	*Centella asiatica*	WXB & QSZ
E5-272B	*Adelencyrtus aulacaspidis* nr2		*Aulacaspis spinosa*	D5-272B	Fujian, Liancheng	2015.5.3	*Centella asiatica*	WXB & QSZ
E5-272C	*Adelencyrtus aulacaspidis* nr2		*Aulacaspis spinosa*	D5-003A	Fujian, Liancheng	2015.5.3	*Centella asiatica*	WXB & QSZ
E5-272D	*Adelencyrtus aulacaspidis* nr2		*Aulacaspis spinosa*	D5-003B	Fujian, Liancheng	2015.5.3	*Centella asiatica*	WXB & QSZ
E5-303A	*Adelencyrtus chinensis*	10	*Unaspis yanonensis*		Guizhou, Guiyang	2015.7.27	*Citrus maxima*	JFW&MMN
E5-303B	*Adelencyrtus chinensis*		*Unaspis yanonensis*	D5-303A D5-303B	Guizhou, Guiyang	2015.7.27	*Citrus maxima*	JFW&MMN
E5-303C	*Adelencyrtus chinensis*		*Unaspis yanonensis*		Guizhou, Guiyang	2015.7.27	*Citrus maxima*	JFW&MMN
7-90	*Epitetracnemus intersectus*	2	*Lepidosaphes ulmi*		Shanxi, Taiyuan	2006.7.16	*Ulmus pumila*	YZZ
E2-063A	*Epitetracnemus intersectus*	9	*Lepidosaphes ulmi*	S2-155A S2-155B	Heilongjiang, Harbin	2012.6.6	*Ulmus pumila*	XWL
E2-063B	*Epitetracnemus intersectus*		*Lepidosaphes ulmi*	S2-155C S2-155D	Heilongjiang, Harbin	2012.6.6	*Ulmus pumila*	XWL
E3-393A	*Epitetracnemus intersectus*	9	*Lepidosaphes ulmi*		Heilongjiang, Harbin	2013.6.8	*Ulmus pumila*	XWL
E3-393B	*Epitetracnemus intersectus*		*Lepidosaphes ulmi*		Heilongjiang, Harbin	2013.6.8	*Ulmus pumila*	XWL
E3-393C	*Epitetracnemus intersectus*		*Lepidosaphes ulmi*		Heilongjiang, Harbin	2013.6.8	*Ulmus pumila*	XWL
E3-391A	*Epitetracnemus intersectus*	8	*Lepidosaphes ulmi*		Qinghai, Xining	2013.5.29	*Salix babylonica*	WXB&HBL&XZ
E3-391B	*Epitetracnemus intersectus*		*Lepidosaphes ulmi*	S3-914	Qinghai, Xining	2013.5.29	*Salix babylonica*	WXB&HBL&XZ
E3-391C	*Epitetracnemus intersectus*		*Lepidosaphes ulmi*	S3-914B	Qinghai, Xining	2013.5.29	*Salix babylonica*	WXB&HBL&XZ
E3-391D	*Epitetracnemus intersectus*		*Lepidosaphes ulmi*		Qinghai, Xining	2013.5.29	*Salix babylonica*	WXB&HBL&XZ
E4-023A	*Epitetracnemus comis* nr1	23	*Pseudaulacaspis pentagona* nr1	SE4-023A	Beijing, Haidian	2014.5.15	*Amygdalus persica*	YW
E4-023B	*Epitetracnemus comis* nr1		*Pseudaulacaspis pentagona* nr1	SE4-023B	Beijing, Haidian	2014.5.15	*Amygdalus persica*	YW
E4-027A	*Epitetracnemus comis* nr2	3	*Pseudaulacaspis pentagona* nr1		Beijing, Haidian	2014.5.15	*Sophora japonica*	YW
E4-027B	*Epitetracnemus comis* nr2		*Pseudaulacaspis pentagona* nr1		Beijing, Haidian	2014.5.15	*Sophora japonica*	YW
E4-027C	*Epitetracnemus comis* nr2		*Pseudaulacaspis pentagona* nr1		Beijing, Haidian	2014.5.15	*Sophora japonica*	YW
E4-137A	*Epitetracnemus comis* nr2	8	*Pseudaulacaspis pentagona* nr1		Sichuan, Yibin	2014.7.20	*Ligustrum lucidum*	WXB&JTZ
E4-137B	*Epitetracnemus comis* nr2		*Pseudaulacaspis pentagona* nr1		Sichuan, Yibin	2014.7.20	*Ligustrum lucidum*	WXB&JTZ
E4-137C	*Epitetracnemus comis* nr2		*Pseudaulacaspis pentagona* nr1	S4-327A S4-327B	Sichuan, Yibin	2014.7.20	*Ligustrum lucidum*	WXB&JTZ
E4-137D	*Epitetracnemus comis* nr2		*Pseudaulacaspis pentagona* nr1		Sichuan, Yibin	2014.7.20	*Ligustrum lucidum*	WXB&JTZ
E4-137E	*Epitetracnemus comis* nr2		*Pseudaulacaspis pentagona* nr1		Sichuan, Yibin	2014.7.20	*Ligustrum lucidum*	WXB&JTZ
E6-165A	*Epitetracnemus comis* nr3	2	*Pseudaulacaspis pentagona* nr3	SE6-165ABC	Yunnan, Mengla	2016.10.12	*Allemanda neriifolia*	YGQ & XBW
E6-165B	*Epitetracnemus comis* nr3		*Pseudaulacaspis pentagona* nr3		Yunnan, Mengla	2016.10.12	*Allemanda neriifolia*	YGQ & XBW
E3-394A	*Zaomma lambinus* nr1	7	*Lepidosaphes ulmi*		Heilongjiang, Harbin	2013.6.8	*Ulmus pumila*	XWL
E3-394B	*Zaomma lambinus* nr1		*Lepidosaphes ulmi*		Heilongjiang, Harbin	2013.6.8	*Ulmus pumila*	XWL
E3-389A	*Zaomma lambinus* nr1	3	*Lepidosaphes ulmi*		Qinghai, Xining	2013.5.29	*Salix babylonica*	WXB&HBL&XZ
E3-389B	*Zaomma lambinus* nr1		*Lepidosaphes ulmi*	S3-914 S3-914B	Qinghai, Xining	2013.5.29	*Salix babylonica*	WXB&HBL&XZ
E3-389C	*Zaomma lambinus* nr1		*Lepidosaphes ulmi*		Qinghai, Xining	2013.5.29	*Salix babylonica*	WXB&HBL&XZ
E5-273A	*Zaomma lambinus* nr1	15	*Aulacaspis spinosa*	D5-272A	Fujian, Liancheng	2015.5.3	*Centella asiatica*	WXB & QSZ
E5-273B	*Zaomma lambinus* nr1		*Aulacaspis spinosa*	D5-272B	Fujian, Liancheng	2015.5.3	*Centella asiatica*	WXB & QSZ
E5-273C	*Zaomma lambinus* nr1		*Aulacaspis spinosa*	D5-003A	Fujian, Liancheng	2015.5.3	*Centella asiatica*	WXB & QSZ
E5-273D	*Zaomma lambinus* nr1		*Aulacaspis spinosa*	D5-003B	Fujian, Liancheng	2015.5.3	*Centella asiatica*	WXB & QSZ
E4-024A	*Zaomma lambinus* nr2	7	*Pseudaulacaspis pentagona* nr1		Beijing, Haidian	2014.5.15	*Amygdalus persica*	YW
E4-024B	*Zaomma lambinus* nr2		*Pseudaulacaspis pentagona* nr1	SE4-023A	Beijing, Haidian	2014.5.15	*Amygdalus persica*	YW
E4-024C	*Zaomma lambinus* nr2		*Pseudaulacaspis pentagona* nr1	SE4-023B	Beijing, Haidian	2014.5.15	*Amygdalus persica*	YW
E4-024D	*Zaomma lambinus* nr2		*Pseudaulacaspis pentagona* nr1		Beijing, Haidian	2014.5.15	*Amygdalus persica*	YW
E4-215A	*Zaomma lambinus* nr3	4	*Pseudaulacaspis pentagona* nr1		Hebei, Chengde	2014.8.9	*Amygdalus persica*	QSZ
E4-215B	*Zaomma lambinus* nr3		*Pseudaulacaspis pentagona* nr1	SE4-215A SE4-215B	Hebei, Chengde	2014.8.9	*Amygdalus persica*	QSZ
E4-215C	*Zaomma lambinus* nr3		*Pseudaulacaspis pentagona* nr1		Hebei, Chengde	2014.8.9	*Amygdalus persica*	QSZ
E6-063A	*Zaomma lambinus* nr4	25	*Aonidiella pini*	SE6-062A	Hainan, Haikou	2016.8.27	*Plumeria rubra*	YGQ
E6-063B	*Zaomma lambinus* nr4		*Aonidiella pini*	SE6-062B	Hainan, Haikou	2016.8.27	*Plumeria rubra*	YGQ
E6-063C	*Zaomma lambinus* nr4		*Aonidiella pini*	SE6-062C	Hainan, Haikou	2016.8.27	*Plumeria rubra*	YGQ
7-6	*Arrhenophagus albitibiae* nr1	31	*Aulacaspis rosae*		Hainan, Diaoluo	2007.5.8	*Cvcas revoluta*	YZZ
E3-361	*Arrhenophagus albitibiae* nr1	1	*Aulacaspis rosae*		Yunnan, Jinghong	2013.10.26	*Cvcas revoluta*	YZZ&QSZ
E5-300A	*Arrhenophagus albitibiae* nr1	521	*Aulacaspis rosae*		Guizhou, Guiyang	2015.7.27	*Cvcas revoluta*	JFW&MMN
E5-300B	*Arrhenophagus albitibiae* nr1		*Aulacaspis rosae*		Guizhou, Guiyang	2015.7.27	*Cvcas revoluta*	JFW&MMN
E5-300C	*Arrhenophagus albitibiae* nr1		*Aulacaspis rosae*	D5-300A D5-300B	Guizhou, Guiyang	2015.7.27	*Cvcas revoluta*	JFW&MMN
E5-300D	*Arrhenophagus albitibiae* nr1		*Aulacaspis rosae*		Guizhou, Guiyang	2015.7.27	*Cvcas revoluta*	JFW&MMN
E5-300E	*Arrhenophagus albitibiae* nr1		*Aulacaspis rosae*		Guizhou, Guiyang	2015.7.27	*Cvcas revoluta*	JFW&MMN
E6-179A	*Arrhenophagus albitibiae* nr1	300	*Aulacaspis yasumatsui* nr1	SE6-179A	Yunnan, Mengla	2016.10.26	*Cycas revoluta*	YGQ & XBW
E6-179B	*Arrhenophagus albitibiae* nr1		*Aulacaspis yasumatsui* nr1	SE6-179B	Yunnan, Mengla	2016.10.26	*Cycas revoluta*	YGQ & XBW
E6-179C	*Arrhenophagus albitibiae* nr1		*Aulacaspis yasumatsui* nr1	SE6-179C	Yunnan, Mengla	2016.10.26	*Cycas revoluta*	YGQ & XBW
E6-181A	*Arrhenophagus albitibiae* nr1	1000	*Aulacaspis yasumatsui* nr1		Yunnan, Mengla	2016.10.26	*Cycas revoluta*	YGQ & XBW
E6-181B	*Arrhenophagus albitibiae* nr1		*Aulacaspis yasumatsui* nr1	SE6-181A	Yunnan, Mengla	2016.10.26	*Cycas revoluta*	YGQ & XBW
E6-181C	*Arrhenophagus albitibiae* nr1		*Aulacaspis yasumatsui* nr1	SE6-181B	Yunnan, Mengla	2016.10.26	*Cycas revoluta*	YGQ & XBW
E6-181D	*Arrhenophagus albitibiae* nr1		*Aulacaspis yasumatsui* nr1	SE6-181C	Yunnan, Mengla	2016.10.26	*Cycas revoluta*	YGQ & XBW
E6-181E	*Arrhenophagus albitibiae* nr1		*Aulacaspis yasumatsui* nr1		Yunnan, Mengla	2016.10.26	*Cycas revoluta*	YGQ & XBW
E5-317A	*Arrhenophagus albitibiae* nr1	60	*Aulacaspis yasumatsui* nr2	D5-317A	Hainan, Danzhou	2015.11.1	*Cycas revoluta*	YGQ
E5-317B	*Arrhenophagus albitibiae* nr1		*Aulacaspis yasumatsui* nr2	D5-317B	Hainan, Danzhou	2015.11.1	*Cycas revoluta*	YGQ
E5-317C	*Arrhenophagus albitibiae* nr1		*Aulacaspis yasumatsui* nr2	D5-317C	Hainan, Danzhou	2015.11.1	*Cycas revoluta*	YGQ
E5-001A	*Arrhenophagus albitibiae* nr1	233	*Aulacaspis yasumatsui* nr3		Fujian, Fuzhou	2015.1.12	*Cycas szechuanensis*	LFP
E5-001B	*Arrhenophagus albitibiae* nr1		*Aulacaspis yasumatsui* nr3	SE5-001A SE5-001B	Fujian, Fuzhou	2015.1.12	*Cycas szechuanensis*	LFP
E5-001C	*Arrhenophagus albitibiae* nr1		*Aulacaspis yasumatsui* nr3		Fujian, Fuzhou	2015.1.12	*Cycas szechuanensis*	LFP
E4-141A	*Arrhenophagus albitibiae* nr1	300	*Aulacaspis yasumatsui* nr3		Fujian, Fuzhou	2014.10.6	*Cvcas revoluta*	LFP
E4-141B	*Arrhenophagus albitibiae* nr1		*Aulacaspis yasumatsui* nr3		Fujian, Fuzhou	2014.10.6	*Cvcas revoluta*	LFP
E4-141C	*Arrhenophagus albitibiae* nr1		*Aulacaspis yasumatsui* nr3	S4-328	Fujian, Fuzhou	2014.10.6	*Cvcas revoluta*	LFP
E4-141D	*Arrhenophagus albitibiae* nr1		*Aulacaspis yasumatsui* nr3		Fujian, Fuzhou	2014.10.6	*Cvcas revoluta*	LFP
E4-141E	*Arrhenophagus albitibiae* nr1		*Aulacaspis yasumatsui* nr3		Fujian, Fuzhou	2014.10.6	*Cvcas revoluta*	LFP
E6-163A	*Arrhenophagus albitibiae* nr1	1300	*Aulacaspis yasumatsui* nr3	SE6-163A	Yunnan, Ruili	2016.10.23	*Cycas revoluta*	YGQ & XBW

E6-163B	*Arrhenophagus albitibiae* nr1		*Aulacaspis yasumatsui* nr3	SE6-163B	Yunnan, Ruili	2016.10.23	*Cycas revoluta*	YGQ & XBW
E6-163C	*Arrhenophagus albitibiae* nr1		*Aulacaspis yasumatsui* nr3	SE6-163C	Yunnan, Ruili	2016.10.23	*Cycas revoluta*	YGQ & XBW
E6-163D	*Arrhenophagus albitibiae* nr1		*Aulacaspis yasumatsui* nr3		Yunnan, Ruili	2016.10.23	*Cycas revoluta*	YGQ & XBW
E6-163E	*Arrhenophagus albitibiae* nr1		*Aulacaspis yasumatsui* nr3		Yunnan, Ruili	2016.10.23	*Cycas revoluta*	YGQ & XBW
E6-163F	*Arrhenophagus albitibiae* nr1		*Aulacaspis yasumatsui* nr3		Yunnan, Ruili	2016.10.23	*Cycas revoluta*	YGQ & XBW
E4-138A	*Arrhenophagus albitibiae* nr2	13	*Pseudaulacaspis pentagona* nr1		Sichuan, Yibin	2014.7.20	*Ligustrum lucidum*	WXB&JTZ
E4-138B	*Arrhenophagus albitibiae* nr2		*Pseudaulacaspis pentagona* nr1	S4-327A	Sichuan, Yibin	2014.7.20	*Ligustrum lucidum*	WXB&JTZ
E4-138C	*Arrhenophagus albitibiae* nr2		*Pseudaulacaspis pentagona* nr1	S4-327B	Sichuan, Yibin	2014.7.20	*Ligustrum lucidum*	WXB&JTZ
8-01	*Arrhenophagus albitibiae* nr2	2	*Pseudaulacaspis pentagona* nr1		Shanghai, Jiangpu park	2008.5.15	*Nerium indicum*	HLL
E5-295A	*Arrhenophagus albitibiae* nr2	60	*Pseudaulacaspis pentagona* nr1		Guizhou, Guiyang	2015.7.27	*Buxus megistophylla*	JFW&MMN
E5-295B	*Arrhenophagus albitibiae* nr2		*Pseudaulacaspis pentagona* nr1		Guizhou, Guiyang	2015.7.27	*Buxus megistophylla*	JFW&MMN
E5-295C	*Arrhenophagus albitibiae* nr2		*Pseudaulacaspis pentagona* nr1	D5-295A<br>D5-295B	Guizhou, Guiyang	2015.7.27	*Buxus megistophylla*	JFW&MMN
E5-295D	*Arrhenophagus albitibiae* nr2		*Pseudaulacaspis pentagona* nr1		Guizhou, Guiyang	2015.7.27	*Buxus megistophylla*	JFW&MMN
E5-295E	*Arrhenophagus albitibiae* nr2		*Pseudaulacaspis pentagona* nr1		Guizhou, Guiyang	2015.7.27	*Buxus megistophylla*	JFW&MMN
E6-205A	*Arrhenophagus albitibiae* nr2	15	*Pseudaulacaspis pentagona* nr1	SE6-205A	Yunnan, Ruili	2016.10.22	*Buxus sinica*	YGQ & XBW
E6-205B	*Arrhenophagus albitibiae* nr2		*Pseudaulacaspis pentagona* nr1	SE6-205B	Yunnan, Ruili	2016.10.22	*Buxus sinica*	YGQ & XBW
E6-205C	*Arrhenophagus albitibiae* nr2		*Pseudaulacaspis pentagona* nr1	SE6-205C	Yunnan, Ruili	2016.10.22	*Buxus sinica*	YGQ & XBW
E3-138	*Arrhenophagus albitibiae* nr3	1	*Pseudaulacaspis cockerelli*		Guangdong, Zhaoqing	2013.4.27	*Ficus concinna*	XZ&WXB
E2-060A	*Arrhenophagus albitibiae* nr3	120	*Pseudaulacaspis cockerelli*		Guangdong, Zhaoqing	2012.4.16	*Ficus concinna*	YW&JD&HBL
E2-060B	*Arrhenophagus albitibiae* nr3		*Pseudaulacaspis cockerelli*		Guangdong, Zhaoqing	2012.4.16	*Ficus concinna*	YW&JD&HBL
E2-060C	*Arrhenophagus albitibiae* nr3		*Pseudaulacaspis cockerelli*	SE2-060A	Guangdong, Zhaoqing	2012.4.16	*Ficus concinna*	YW&JD&HBL
E2-060D	*Arrhenophagus albitibiae* nr3		*Pseudaulacaspis cockerelli*	SE2-060B	Guangdong, Zhaoqing	2012.4.16	*Ficus concinna*	YW&JD&HBL
E2-060E	*Arrhenophagus albitibiae* nr3		*Pseudaulacaspis cockerelli*		Guangdong, Zhaoqing	2012.4.16	*Ficus concinna*	YW&JD&HBL
E2-060F	*Arrhenophagus albitibiae* nr3		*Pseudaulacaspis cockerelli*		Guangdong, Zhaoqing	2012.4.16	*Ficus concinna*	YW&JD&HBL
E5-107A	*Arrhenophagus albitibiae* nr3	2	*Pseudaulacaspis cockerelli*	D5-001A	Guangxi, Beihai	2015.5.4	*Sedum lineare*	QSZ & WXB
E5-107B	*Arrhenophagus albitibiae* nr3		*Pseudaulacaspis cockerelli*	D5-001B	Guangxi, Beihai	2015.5.4	*Sedum lineare*	QSZ & WXB
E5-054A	*Arrhenophagus albitibiae* nr3	18	*Pseudaulacaspis cockerelli*		Guangdong, Guangzhou	2015.4.11	*Ligustrum compactum*	XZ
E5-054B	*Arrhenophagus albitibiae* nr3		*Pseudaulacaspis cockerelli*		Guangdong, Guangzhou	2015.4.11	*Ligustrum compactum*	XZ
E5-081A	*Arrhenophagus albitibiae* nr3	1	*Pseudaulacaspis cockerelli*	SE5-080	Shanghai, Changning	2015.4.17	*Trachycarpus fortunei*	HLL
E5-306A	*Arrhenophagus albitibiae* nr3	109			Guizhou, Duyun	2015.7.30	*Citrus reticulata*	WJF
E5-306B	*Arrhenophagus albitibiae* nr3				Guizhou, Duyun	2015.7.30	*Citrus reticulata*	WJF
E5-306C	*Arrhenophagus albitibiae* nr3				Guizhou, Duyun	2015.7.30	*Citrus reticulata*	WJF
E4-133A	*Arrhenophagus albitibiae* nr4	26	*Fiorinia pinicola*		Hainan, Wanning	2014.7.23	*Iris tectorum*	BC
E4-133B	*Arrhenophagus albitibiae* nr4		*Fiorinia pinicola*		Hainan, Wanning	2014.7.23	*Iris tectorum*	BC
E4-133C	*Arrhenophagus albitibiae* nr4		*Fiorinia pinicola*	S4-318	Hainan, Wanning	2014.7.23	*Iris tectorum*	BC
E4-133D	*Arrhenophagus albitibiae* nr4		*Fiorinia pinicola*		Hainan, Wanning	2014.7.23	*Iris tectorum*	BC
E4-133E	*Arrhenophagus albitibiae* nr4		*Fiorinia pinicola*		Hainan, Wanning	2014.7.23	*Iris tectorum*	BC
E5-015A	*Arrhenophagus albitibiae* nr5	60	*Pseudaulacaspis cockerelli*		Yunnan, Yuanjiang	2015.1.12	*Fagraea ceilanica*	XZ
E5-015B	*Arrhenophagus albitibiae* nr5		*Pseudaulacaspis cockerelli*		Yunnan, Yuanjiang	2015.1.12	*Fagraea ceilanica*	XZ
E5-015C	*Arrhenophagus albitibiae* nr5		*Pseudaulacaspis cockerelli*		Yunnan, Yuanjiang	2015.1.12	*Fagraea ceilanica*	XZ
E5-015D	*Arrhenophagus albitibiae* nr5		*Pseudaulacaspis cockerelli*		Yunnan, Yuanjiang	2015.1.12	*Fagraea ceilanica*	XZ
E5-015E	*Arrhenophagus albitibiae* nr5		*Pseudaulacaspis cockerelli*		Yunnan, Yuanjiang	2015.1.12	*Fagraea ceilanica*	XZ
E6-166A	*Arrhenophagus albitibiae* nr6	3	*Pseudaulacaspis pentagona* nr3	SE6-165A<br>SE6-165B	Yunnan, Mengla	2016.10.12	*Allemanda neriifolia*	YGQ & XBW
E6-166B	*Arrhenophagus albitibiae* nr6		*Pseudaulacaspis pentagona* nr3	SE6-165C	Yunnan, Mengla	2016.10.12	*Allemanda neriifolia*	YGQ & XBW
E5-014A	*Thomsonisca amathus* nr1	90	*Pseudaulacaspis cockerelli*		Yunnan, Yuanjiang	2015.2.27	*Fagraea ceilanica*	XZ
E5-014B	*Thomsonisca amathus* nr1		*Pseudaulacaspis cockerelli*		Yunnan, Yuanjiang	2015.2.27	*Fagraea ceilanica*	XZ
E5-014C	*Thomsonisca amathus* nr1		*Pseudaulacaspis cockerelli*		Yunnan, Yuanjiang	2015.2.27	*Fagraea ceilanica*	XZ
E5-014D	*Thomsonisca amathus* nr1		*Pseudaulacaspis cockerelli*		Yunnan, Yuanjiang	2015.2.27	*Fagraea ceilanica*	XZ
E5-014E	*Thomsonisca amathus* nr1		*Pseudaulacaspis cockerelli*		Yunnan, Yuanjiang	2015.2.27	*Fagraea ceilanica*	XZ
E5-053A	*Thomsonisca amathus* nr1	13	*Pseudaulacaspis cockerelli*		Guangdong, Guangzhou	2015.4.11	*Ligustrum compactum*	XZ
E5-053B	*Thomsonisca amathus* nr1		*Pseudaulacaspis cockerelli*		Guangdong, Guangzhou	2015.4.11	*Ligustrum compactum*	XZ
E5-080A	*Thomsonisca amathus* nr1	9	*Pseudaulacaspis cockerelli*		Shanghai, Changning	2015.4.17	*Trachycarpus fortunei*	HLL
E5-080B	*Thomsonisca amathus* nr1		*Pseudaulacaspis cockerelli*	SE5-080	Shanghai, Changning	2015.4.17	*Trachycarpus fortunei*	HLL
E5-080C	*Thomsonisca amathus* nr1		*Pseudaulacaspis cockerelli*		Shanghai, Changning	2015.4.17	*Trachycarpus fortunei*	HLL
E5-082A	*Thomsonisca amathus* nr1	13	*Pseudaulacaspis cockerelli*	SE5-082	Shanghai, Xuhui	2015.4.17	*Aucuba japonica*	HLL
E5-082B	*Thomsonisca amathus* nr1		*Pseudaulacaspis cockerelli*		Shanghai, Xuhui	2015.4.17	*Aucuba japonica*	HLL
E5-084A	*Thomsonisca amathus* nr1	3	*Pseudaulacaspis cockerelli*	SE5-084	Shanghai, Xuhui	2015.4.17	*Hedera nepalensis*	HLL
E5-106A	*Thomsonisca amathus* nr1	3	*Pseudaulacaspis cockerelli*		Guangxi, Beihai	2015.5.4	*Sedum lineare*	QSZ & WXB
E5-106B	*Thomsonisca amathus* nr1		*Pseudaulacaspis cockerelli*	D5-001A<br>D5-001B	Guangxi, Beihai	2015.5.4	*Sedum lineare*	QSZ & WXB
E5-106C	*Thomsonisca amathus* nr1		*Pseudaulacaspis cockerelli*		Guangxi, Beihai	2015.5.4	*Sedum lineare*	QSZ & WXB
E5-223A	*Thomsonisca amathus* nr2	1	*Pseudaulacaspis pentagona* nr2	D5-222A<br>D5-222B	Guangdong, Heyuan	2015.5.3	*Nerium indicum*	WXB & QSZ
E5-297A	*Thomsonisca amathus* nr3	8	*Lepidosaphes gloverii*		Guizhou, Guiyang	2015.7.27	*Buxus megistophylla*	JFW & MMN
E5-297B	*Thomsonisca amathus* nr3		*Lepidosaphes gloverii*	D5-297A<br>D5-297B	Guizhou, Guiyang	2015.7.27	*Buxus megistophylla*	JFW & MMN
E5-297C	*Thomsonisca amathus* nr3		*Lepidosaphes gloverii*		Guizhou, Guiyang	2015.7.27	*Buxus megistophylla*	JFW & MMN
E5-222A	*Adelencyrtoides* sp.	45	*Pseudaulacaspis pentagona* nr2		Guangdong, Heyuan	2015.5.3	*Nerium indicum*	WXB & QSZ
E5-222B	*Adelencyrtoides* sp.		*Pseudaulacaspis pentagona* nr2	D5-222A<br>D5-222B	Guangdong, Heyuan	2015.5.3	*Nerium indicum*	WXB & QSZ
E5-222C	*Adelencyrtoides* sp.		*Pseudaulacaspis pentagona* nr2		Guangdong, Heyuan	2015.5.3	*Nerium indicum*	WXB & QSZ
E5-279A	*Adelencyrtoides* sp.	4	*Pseudaulacaspis pentagona* nr2		Fujian, Xiamen	2015.5.15	*Nerium indicum*	WXB & QSZ
E5-279B	*Adelencyrtoides* sp.		*Pseudaulacaspis pentagona* nr2	SE5-278A<br>SE5-278B	Fujian, Xiamen	2015.5.15	*Nerium indicum*	WXB & QSZ
E5-279C	*Adelencyrtoides* sp.		*Pseudaulacaspis pentagona* nr2		Fujian, Xiamen	2015.5.15	*Nerium indicum*	WXB & QSZ
E6-204A	*Adelencyrtoides* sp.	4	*Pseudaulacaspis pentagona* nr1	SE6-205A<br>SE6-205B<br>SE6-205C	Yunnan, Ruili	2016.10.22	*Buxus sinica*	YGQ & XBW
E4-026A	*Coccidencyrtus steinbergi* nr1	5	*Diaspidiotus perniciosus*		Beijing, Haidian	2014.5.15	*Ulmus pumila*	YW
E4-026B	*Coccidencyrtus steinbergi* nr1		*Diaspidiotus perniciosus*		Beijing, Haidian	2014.5.15	*Ulmus pumila*	YW
E5-153A	*Coccidencyrtus steinbergi* nr1	15	*Diaspidiotus perniciosus*	D5-002A	Beijing, Badachu	2015.5.17	*Ulmus pumila*	YGQ
E5-153B	*Coccidencyrtus steinbergi* nr1		*Diaspidiotus perniciosus*	D5-002B	Beijing, Badachu	2015.5.17	*Ulmus pumila*	YGQ
E5-153C	*Coccidencyrtus steinbergi* nr1		*Diaspidiotus perniciosus*	D5-002C	Beijing, Badachu	2015.5.17	*Ulmus pumila*	YGQ
E5-171A	*Coccidencyrtus steinbergi* nr1	15	*Diaspidiotus perniciosus*		Beijing, Haidina	2015.5.23	*Ulmus pumila*	YZZ
E5-171B	*Coccidencyrtus steinbergi* nr1		*Diaspidiotus perniciosus*	D5-171A<br>D5-171B	Beijing, Haidina	2015.5.23	*Ulmus pumila*	YZZ
E5-171C	*Coccidencyrtus steinbergi* nr1		*Diaspidiotus perniciosus*		Beijing, Haidina	2015.5.23	*Ulmus pumila*	YZZ
E5-251A	*Coccidencyrtus steinbergi* nr2	45	*Aulacaspis tubercularis* nr2		Jiangxi, Dingnan	2015.5.3	*Cinnamomum pedunculatum*	WXB & QSZ
E5-251B	*Coccidencyrtus steinbergi* nr2		*Aulacaspis tubercularis* nr2		Jiangxi, Dingnan	2015.5.3	*Cinnamomum pedunculatum*	WXB & QSZ
E5-251C	*Coccidencyrtus steinbergi* nr2		*Aulacaspis tubercularis* nr2	D5-251A<br>D5-251B	Jiangxi, Dingnan	2015.5.3	*Cinnamomum pedunculatum*	WXB & QSZ
E5-251D	*Coccidencyrtus steinbergi* nr2		*Aulacaspis tubercularis* nr2		Jiangxi, Dingnan	2015.5.3	*Cinnamomum pedunculatum*	WXB & QSZ
E5-251E	*Coccidencyrtus steinbergi* nr2		*Aulacaspis tubercularis* nr2		Jiangxi, Dingnan	2015.5.3	*Cinnamomum pedunculatum*	WXB & QSZ
E6-185A	*Coccidencyrtus steinbergi* nr2	3	*Aulacaspis tubercularis* nr2	SE6-185A	Yunnan, Lanchang	2016.10.12	*Cinnamomum japonicum*	YGQ & XBW
E6-185B	*Coccidencyrtus steinbergi* nr2		*Aulacaspis tubercularis* nr2	SE6-185B	Yunnan, Lanchang	2016.10.12	*Cinnamomum japonicum*	YGQ & XBW
E6-185C	*Coccidencyrtus steinbergi* nr2		*Aulacaspis tubercularis* nr2	SE6-185C	Yunnan, Lanchang	2016.10.12	*Cinnamomum japonicum*	YGQ & XBW

E5-304A	*Coccidencyrtus steinbergi* nr3	4	*Lepidosaphes pinnaeformis* nr1	D5-304A	Guizhou, Kaili	2015.7.30	*Magnolia denudata*	JFW
E5-304B	*Coccidencyrtus steinbergi* nr3		*Lepidosaphes pinnaeformis* nr1	D5-304B	Guizhou, Kaili	2015.7.30	*Magnolia denudata*	JFW
E6-201A	*Coccidencyrtus steinbergi* nr4	30	*Pinnaspis* sp.		Yunnan, Baoshan	2016.10.12	*Erythrina crista-gall*	YGQ & XBW
E6-201B	*Coccidencyrtus steinbergi* nr4		*Pinnaspis* sp.	SE6-201A	Yunnan, Baoshan	2016.10.12	*Erythrina crista-gall*	YGQ & XBW
E6-201C	*Coccidencyrtus steinbergi* nr4		*Pinnaspis* sp.	SE6-201B	Yunnan, Baoshan	2016.10.12	*Erythrina crista-gall*	YGQ & XBW
E6-201D	*Coccidencyrtus steinbergi* nr4		*Pinnaspis* sp.	SE6-201C	Yunnan, Baoshan	2016.10.12	*Erythrina crista-gall*	YGQ & XBW
E6-201E	*Coccidencyrtus steinbergi* nr4		*Pinnaspis* sp.		Yunnan, Baoshan	2016.10.12	*Erythrina crista-gall*	YGQ & XBW
E5-305A	*Coccidencyrtus steinbergi* nr5	10	*Lepidosaphes pinnaeformis* nr2	D5-305A	Guizhou, Duyun	2015.7.30	*Cinnamomum camphora*	WJF
E5-305B	*Coccidencyrtus steinbergi* nr5		*Lepidosaphes pinnaeformis* nr2	D5-305B D5-305C	Guizhou, Duyun	2015.7.30	*Cinnamomum camphora*	WJF
E5-305C	*Coccidencyrtus steinbergi* nr5		*Lepidosaphes pinnaeformis* nr2	D5-305D	Guizhou, Duyun	2015.7.30	*Cinnamomum camphora*	WJF
08-47	*Coccidencyrtus steinbergi* nr6	1			Chongqing	2009.6.17	Fig	DL
07-112A	*Coccidencyrtus steinbergi* nr6	5			Shanxi, Taiyuan	2007.6.14	*Zizyphus jujuba*	ZYZ
07-112B	*Coccidencyrtus steinbergi* nr6				Shanxi, Taiyuan	2007.6.14	*Zizyphus jujuba*	ZYZ
07-112C	*Coccidencyrtus steinbergi* nr6				Shanxi, Taiyuan	2007.6.14	*Zizyphus jujuba*	ZYZ
E4-135A	*Plagiomerus* sp.	10	*Aulacaspis* sp.		Sichuan, Langzhong	2014.7.23	*Citrus maxima*	WXB&JTZ
E4-135B	*Plagiomerus* sp.		*Aulacaspis* sp.		Sichuan, Langzhong	2014.7.23	*Citrus maxima*	WXB&JTZ
E4-135C	*Plagiomerus* sp.		*Aulacaspis* sp.		Sichuan, Langzhong	2014.7.23	*Citrus maxima*	WXB&JTZ
E4-135D	*Plagiomerus* sp.		*Aulacaspis* sp.		Sichuan, Langzhong	2014.7.23	*Citrus maxima*	WXB&JTZ
E4-135E	*Plagiomerus* sp.		*Aulacaspis* sp.		Sichuan, Langzhong	2014.7.23	*Citrus maxima*	WXB&JTZ
E2-205	*Plagiomerus* sp.	1	*Aulacaspis* sp.		Hunan, Zhangjiajie	2012.9.25		
E5-283A	*Plagiomerus* sp.	1	*Aulacaspis tubercularis* nr1	SE5-283A SE5-283B	Zhejiang, Wenzhou	2015.5.20	*Cinnamomum camphora*	WXB & QSZ
E5-342A	*Plagiomerus* sp.	4	*Aulacaspis tubercularis* nr1	D5-342A	Hunan, Xiangtan	2015.10.26	*Cinnamomum camphora*	YGQ & JFW
E5-342B	*Plagiomerus* sp.		*Aulacaspis tubercularis* nr1	D5-342B	Hunan, Xiangtan	2015.10.26	*Cinnamomum camphora*	YGQ & JFW
E5-342C	*Plagiomerus* sp.		*Aulacaspis tubercularis* nr1		Hunan, Xiangtan	2015.10.26	*Cinnamomum camphora*	YGQ & JFW
E4-136A	*Caenohomalopoda shikokuensis*	5	*Odonaspis secreta*		Sichuan, Yibin	2014.7.24	*Bambusoideae*	WXB&JTZ
E4-136B	*Caenohomalopoda shikokuensis*		*Odonaspis secreta*		Sichuan, Yibin	2014.7.24	*Bambusoideae*	WXB&JTZ
E4-136C	*Caenohomalopoda shikokuensis*		*Odonaspis secreta*		Sichuan, Yibin	2014.7.24	*Bambusoideae*	WXB&JTZ
E4-136D	*Caenohomalopoda shikokuensis*		*Odonaspis secreta*		Sichuan, Yibin	2014.7.24	*Bambusoideae*	WXB&JTZ
E5-088A	*Caenohomalopoda shikokuensis*	6	*Odonaspis secreta*		Shanghai, Xuhui	2015.4.10	*Bambusa multiplex*	HLL
E5-088B	*Caenohomalopoda shikokuensis*		*Odonaspis secreta*		Shanghai, Xuhui	2015.4.10	*Bambusa multiplex*	HLL
E5-088C	*Caenohomalopoda shikokuensis*		*Odonaspis secreta*		Shanghai, Xuhui	2015.4.10	*Bambusa multiplex*	HLL
E5-257A	*Caenohomalopoda shikokuensis*	4	*Odonaspis secreta*	D5-257A	Jiangxi, Ganzhou	2015.5.4	*Bambusa multiplex*	WXB & QSZ
E5-257B	*Caenohomalopoda shikokuensis*		*Odonaspis secreta*	D5-257B	Jiangxi, Ganzhou	2015.5.4	*Bambusa multiplex*	WXB & QSZ
E5-257C	*Caenohomalopoda shikokuensis*		*Odonaspis secreta*		Jiangxi, Ganzhou	2015.5.4	*Bambusa multiplex*	WXB & QSZ
E5-205A	*Caenohomalopoda guamensis*	2	*Odonaspis* sp2	SE5-205A SE5-205B SE5-205C	Guangdong, Zhongshan	2015.5.6	*Bambusa ventricosa*	ZQS & WXB
E5-205B	*Caenohomalopoda guamensis*		*Odonaspis* sp2	SE5-205D SE5-205E	Guangdong, Zhongshan	2015.5.6	*Bambusa ventricosa*	ZQS & WXB
E4-107A	*Anthemus aspidioti*	112	*Diaspidiotus gigas* nr1		Xinjiang, Wulumuqi	2014.5.28	*Populus* sp.	XLT
E4-107B	*Anthemus aspidioti*		*Diaspidiotus gigas* nr1	S4-093	Xinjiang, Wulumuqi	2014.5.28	*Populus* sp.	XLT
E4-107C	*Anthemus aspidioti*		*Diaspidiotus gigas* nr1	S4-094	Xinjiang, Wulumuqi	2014.5.28	*Populus* sp.	XLT
E4-107D	*Anthemus aspidioti*		*Diaspidiotus gigas* nr1		Xinjiang, Wulumuqi	2014.5.28	*Populus* sp.	XLT
E4-107E	*Anthemus aspidioti*		*Diaspidiotus gigas* nr1		Xinjiang, Wulumuqi	2014.5.28	*Populus* sp.	XLT

(Collectors' names are abbreviated as follows: BC = Bo Cai; FY = Feng Yuan; HBL = Hai-Bin Li; HLL = Hong-Liang Li; JD = Jun Deng; JFW = Jiu-Feng Wei; JTZ =Jiang-Tao Zhang; LFP = Ling-Fei Peng; MMN = Min-Min Niu; QSZ = Qing-Song Zhou; XLT = Xiu-Li Tang; XWL = Xiu-Wei Liu; XZ = Xu Zhang; YGQ = Yao-Guang Qin; YW = Ying Wang; YZZ = Yan-Zhou Zhang.)（In the text and figures, abbreviation of provinces are used as follow: Beijing=BJ; Chongqing =CQ; Fujian=FJ; Guangdong=GD; Guangxi=GX; Guizhou=GZ; Hainan=HaN; Hebei=HeB; Heilongjiang=HLJ; Hubei=HuB; Hunan=HuN; Inner Mengolia=IM; Jiangsu=JS; Jiangxi=JX; Qinghai=QH; Shanghai=SH; Shanxi=SX; Shaanxi=ShX; Sichuan=SC; Xinjiang=XJ; Yunnan=YN; Zhejiang=ZJ)
