# Supplementary material for: Host specificity of parasitoids (Encyrtidae) toward armored scale insects (Diaspididae): Untangling the effect of cryptic species on quantitative food webs
Source: Ecol Evol. 2018 Jul 13;8(16):7879–93. doi: 10.1002/ece3.4344 (PMC6144978; doi:10.1002/ece3.4344)
Supplement: Supplementary file 5 [file ECE3-8-7879-s005.docx]

**Morphological Taxonomy**

Specimens studied here included host Diaspididae by collecting infested plant samples in the field and parasitoid Encyrtidae reared from these armoured scale insect. The parasitoids emerged and were killed and preserved in 95% ethanol.

Permanent slide mounts of host adult females from the samples were made according to Henderson (2011) and parasitoid were prepared using a standard procedure modified from Noyes (1982), and deposited in the National Zoological Museum of China, Institute of Zoology, Chinese Academy of Sciences, Beijing.

Assignment of host morphospecies is performed by JFW using specialist literature (Ben-Dov, 1988; Takagi, 1960; Takagi, 1969; Takagi, 1970; Tang, 1977; Tang, 1984) and recognizing 28 host morph-species.

*Aonidiella aurantii Aonidiella citrina*

**

*Aonidiella pini Chrysomphalus aonidum*

*Chrysomphalus bifasciculatus* *Chrysomphalus* sp.

*Diaspidiotus gigas Diaspidiotus* sp.

*Aspidiotus nerii Pseudaonidia duplex*

**

*Aulacaspis rosae Aulacaspis spinosa*

**

*Aulacaspis tubercularis Aulacaspis* sp.

**

*Pseudaulacaspis cockerelli Pseudaulacaspis pentagona*

**

*Lepidosaphes ulmi Lepidosaphes pinnaeformis*

**

*Lepidosaphes* sp. *Unaspis yanonensis*

*Fiorinia pinicola Odonaspis secreta*

Morphological identification of parasitoid is done by co-authors YZZ using specialist literature (Alam, 1972; Chumakova,1964; Fatima & Shafee, 1994; Fonscolombe & Boyer, 1832; Girault, 1915; Gordh & Trjapitzin, 1979; Hayat, 1977; Hayat, Alam & Agarwal, 1975; Noyes, 1988; Noyes & Hui, 1987; Prinsloo, 1979; Prinsloo, 1996; Shafee, Alam & Agarwal, 1975; Sharipov, 1980; Tachikawa, 1956; and Walker, 1839) and recognizing 18 parasitoid morpho-species.


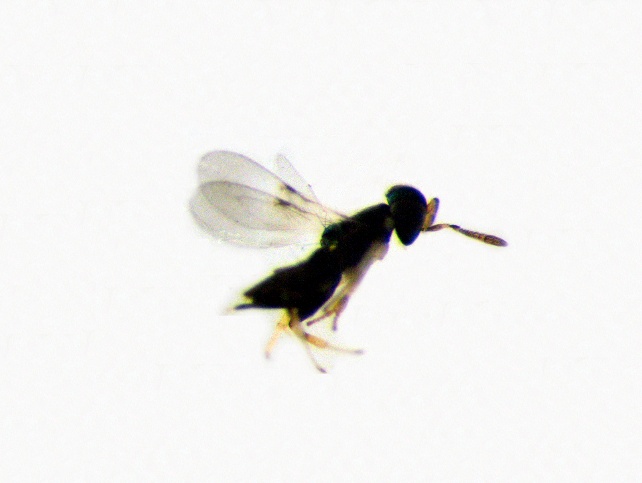

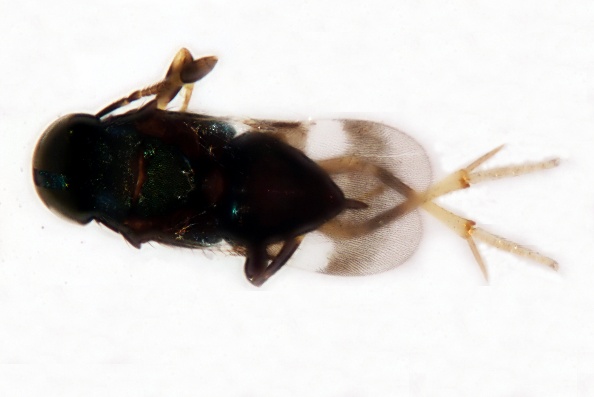


*Adelencyrtus aulacaspidis Adelencyrtus bifasciatus*

*
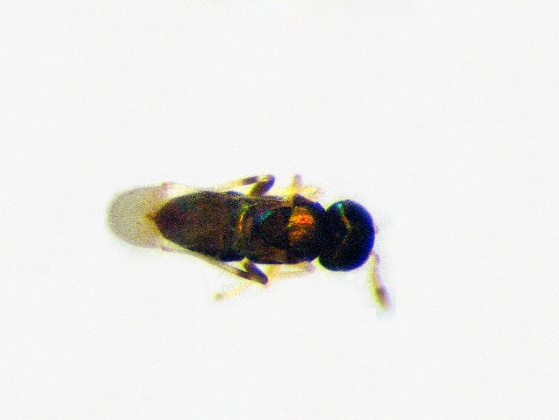

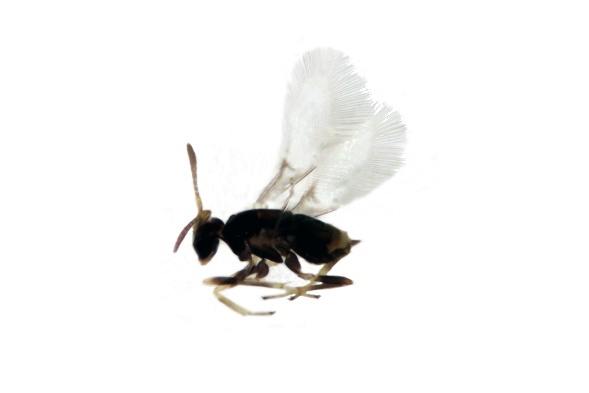
*

*Adelencyrtus* sp. *Anthemus aspidioti*


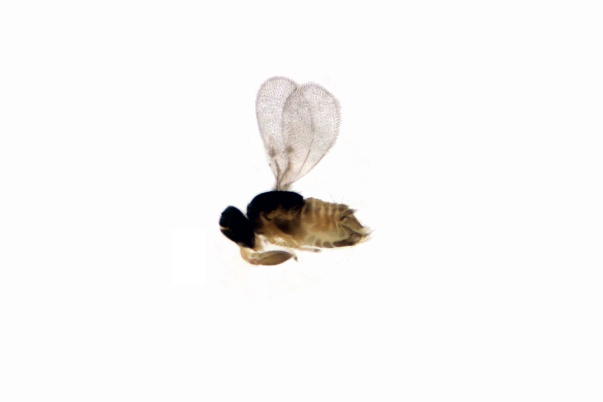

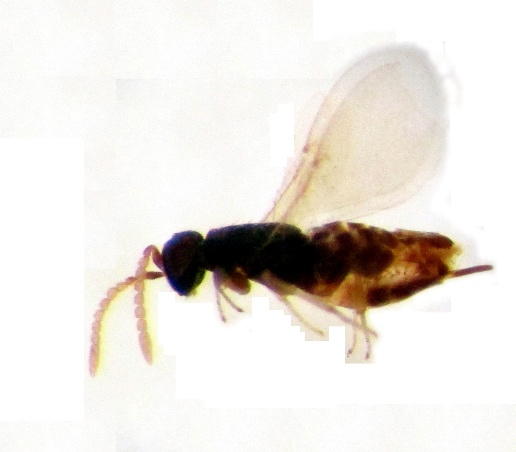


*Arrhenophagus albitibiae Coccidencyrtus steinbergi*

*
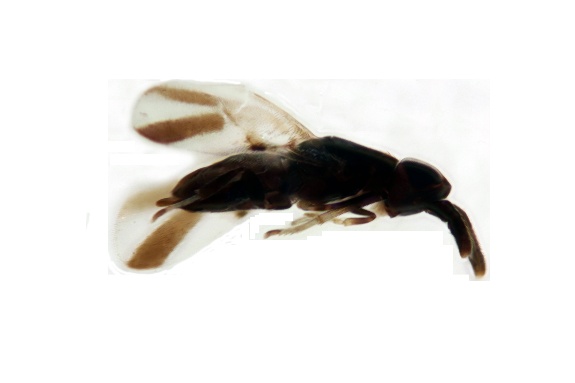

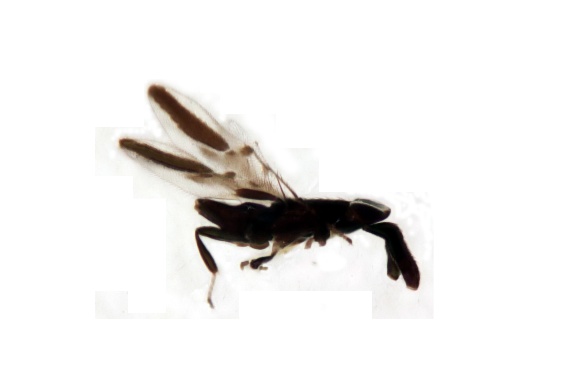
*

*comperiella bifasciata Comperiella indica*

*
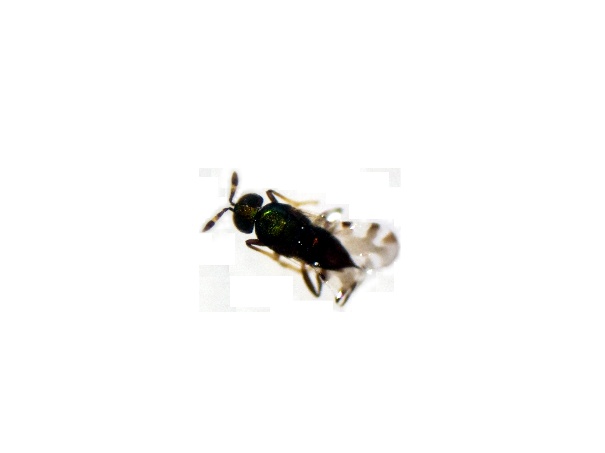

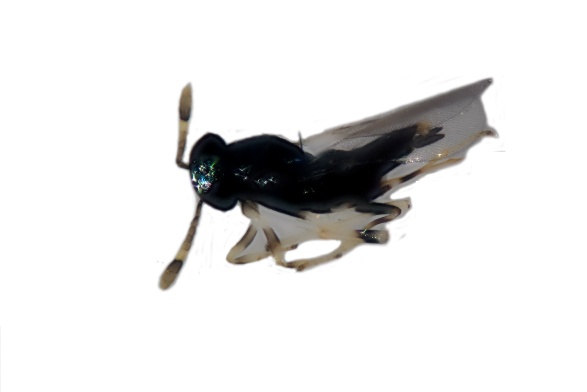
*

*Epitetracnemus intersectus Plagiomerus* sp.


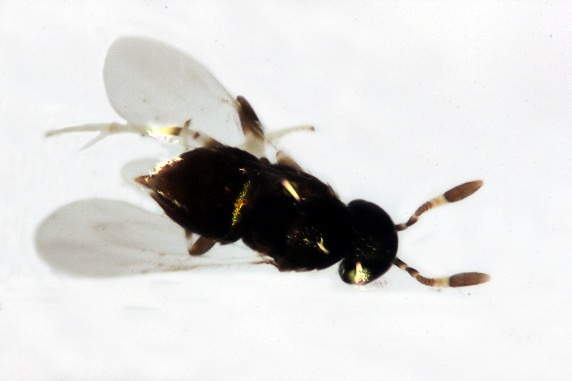

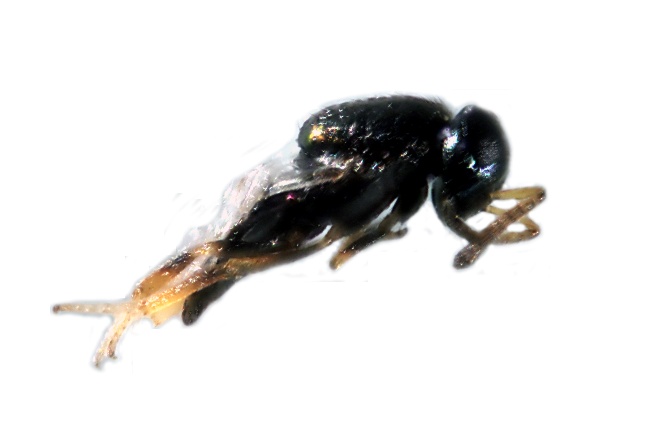


*Zaomma lambinus Adelencyrtoides* sp.

References

Alam, S. M. (1972). New species of Encyrtidae (Hymenoptera: Chalcidoidea) recorded from India. Bulletin of Entomology. *Entomological Society of India*, *11*(2):131-137(479)

Ben-Dov, Y. (1988). A taxonomic analysis of the armored scale tribe Odonaspidini of the world (Homoptera: Coccoidea: Diaspididae). United States Department of Agriculture Technical Bulletin No. 1723, 142 pp.

Chumakova, B. M. (1964). The San José scale, *Diaspidiotus perniciosus* Comst. (Coccidae, Diaspididae), and its parasites in the Soviet Far East. *Entomologicheskoe Obozrenie*, *43*(3), 535-552.

Fatima, A., & Shafee, S. A. (1994). Studies on the taxonomy of Indian encyrtids (Hymenoptera: Encyrtidae). Aligarh Muslim University Publication, Zoological Series on Indian Insect Types. *15*:141pp. (10568)

Fonscolombe, E. L. J. H., & Boyer, D. E. J. H. (1832). Monographia chalciditum galloprovinciae circa aquas degentum. *Annales des Sciences Naturelles* (1) (Zoologie), *26*, 273-307.

Girault, A. A. (1915). Two new species of *Arrhenophagus* with remarks. *Journal of the New York Entomological Society*, *23*(4), 241-242.

Gordh, G., & Trjapitzin, V. A. (1979). Notes on the genus *Zaomma* Ashmead, with a key to species (Hymenoptera: Encyrtidae). *Pan-Pacific Entomologist*. *55*(1):34-40. (13299)

Hayat, M. (1977). Notes on Indian species of *Comperiella* (Hymenoptera: Encyrtidae). *Oriental Insects*. *11*(2):243-250. (14940)

Hayat, M., Alam, M., & Agarwal, M. M. (1975). Taxonomic survey of encyrtid parasites (Hymenoptera: Encyrtidae) in India. *Aligarh Muslim University Publication, Zoological Series on Indian Insect Types*. *9*: iii+112pp. (14991)

Noyes, J. S. (1982). Noyes, j. s. collecting and preserving chalcid wasps (hymenoptera: chalcidoidea). j. nat. hist. *Journal of Natural History*,16(3), 315-334.

Noyes, J. S., & Hui, R. (1987). Two new species of Encyrtidae (Hymenoptera) reared from diaspidid scale insects (Homoptera: Diaspididae) in China. *Entomotaxonomia*, *9*(3), 167-174.

Noyes, J. S. (1988). Encyrtidae (Insecta: Hymenoptera). *Fauna of New Zealand*, *13*.

Henderson, R.C. (2011). Diaspididae (Insecta: Hemiptera: Coccoidea). *Fauna of New Zealand* 66. Manaaki Whenua Press, Lincoln, Canterbury, 275 pp.

Prinsloo, G. L. (1979). On the species of *Zaomma* Ashmead (Hymenoptera: Encyrtidae) from the Ethiopian region. *Journal of the Entomological Society of Southern Africa*. *42*(1):65-75.

Prinsloo, G. L. 1996. The genus *Comperiella* Howard (Hymenoptera: Encyrtidae) in southern Africa: parasitoids of armoured scale insects (Homoptera: Diaspididae). *African Entomology*. *4*(2):153-160.

Shafee, S. A., Alam, M., & Agarwal, M. M. (1975). Taxonomic survey of encyrtid parasites (Hymenoptera: Encyrtidae) in India. *Aligarh Muslim University Publication, Zoological Series on Indian Insect Types*. *10*: iii+125pp.

Sharipov, M. (1980). The encyrtid Anthemus aspidioti Nikolskaya, 1952 (Hymenoptera, Encyrtidae)-a parasite of scale insects (Homoptera, Diaspididae) in Central Asia and Kazakhstan. *Entomologicheskoe Obozrenie*, *59*(2), 381-384.

Tachikawa, T. (1956). Description of a new species of the genus *Pseudhomalopoda* Cirault from Japan, with a list of the known species and their hosts of the *Habrolepis*-like genera (Hymenoptera: Encyrtidae). *Insecta Matsumurana*, *20*(3-4), 90-96.

Takagi, S. (1960) A contribution to the knowledge of the Diaspidini of Japan (Homoptera: Coccoidea) Pt. 1. *Insecta Matsumurana* *23*: 67-100.

Takagi, S. (1969) Diaspididae of Taiwan based on material collected in connection with the Japan-U.S. Co-operative Science Programme, 1965 (Homoptera: Coccoidea). Part I. *Insecta Matsumurana* *32*: 1-110.

Takagi, S. (1970) Diaspididae of taiwan based on material collected in connection with the japan-u.s. co-operative science programme, 1965. (homoptera:coccoidae). part ii. *Insecta Matsumurana* *33*: 1-146.

Tang, F. T. (1977) [The scale insects of horticulture and forest of China. Vol. I.] The Institute of Gardening, Forestry Science of Shenyang. Liaoning, China 259 pp.

Tang, F. T. (1984) [The scale insects of horticulture and forests of China.]. Shanxi Agricultural University Press Research Publication 2: 1-115

Walker, F. (1839). Monographia Chalciditum (Vol. 1). *Bailliere*.
